# SIRT1 ISGylation accelerates tumor progression by unleashing SIRT1 from the inactive state to promote its deacetylase activity

**DOI:** 10.1038/s12276-024-01194-2

**Published:** 2024-03-05

**Authors:** Ji An Kang, Yoon Jung Kim, Kyu Yun Jang, Hye Won Moon, Haeseung Lee, Seonjeong Lee, Hyun Kyu Song, Sang Woo Cho, Yoon Sun Yoo, Hye Gyeong Han, Min-Ju Kim, Myoung Ja Chung, Cheol Yong Choi, Cheolju Lee, Chaeuk Chung, Gang Min Hur, You-Sun Kim, Young Joo Jeon

**Affiliations:** 1 Department of Biochemistry, Chungnam National University College of Medicine, Daejeon, 35015 Republic of Korea; 2 Brain Korea 21 FOUR Project for Medical Science, Chungnam National University, Daejeon, 35015 Republic of Korea; 3 Department of Medical Science, Chungnam National University College of Medicine, Daejeon, 35015 Republic of Korea; 4 Department of Pathology, Jeonbuk National University Medical School, Research Institute of Clinical Medicine of Jeonbuk National University-Biomedical Research Institute of Jeonbuk National University Hospital and Research Institute for Endocrine Sciences, Jeonju, 54896 Republic of Korea; 5 College of Pharmacy, Pusan National University, Busan, 46241 Republic of Korea; 6 Chemical & Biological Integrative Research Center, Korea Institute of Science and Technology, Seoul, 02792 Republic of Korea; 7 Division of Bio-Medical Science & Technology, KIST School, University of Science and Technology, Seoul, 02792 Republic of Korea; 8 Department of Life Sciences, Korea University, Seoul, 02841 Republic of Korea; 9 Department of Biological Sciences, Sungkyunkwan University, Suwon, 16419 Republic of Korea; 10 Division of Pulmonology and Critical Care Medicine, Department of Internal Medicine, College of Medicine, Chungnam National University, Daejeon, 34134 Republic of Korea; 11 Department of Pharmacology, Chungnam National University College of Medicine, Daejeon, 35015 Republic of Korea; 12 Department of Biochemistry, Ajou University, School of Medicine & Department of Biomedical Sciences, Graduate School, Ajou University, Suwon, 16499 Republic of Korea

**Keywords:** Oncogenes, Ubiquitylation

## Abstract

ISG15 is an interferon-stimulated ubiquitin-like protein (UBL) with multifaceted roles as a posttranslational modifier in ISG15 conjugation (ISGylation). However, the mechanistic consequences of ISGylation in cancer have not been fully elucidated, largely due to a lack of knowledge on the ISG15 target repertoire. Here, we identified SIRT1, a nicotinamide adenine dinucleotide (NAD^+^)-dependent protein deacetylase, as a new target for ISGylation. SIRT1 ISGylation impairs the association of SIRT1 with its negative regulator, deleted in breast cancer 1 (DBC1), which unleashes SIRT1 from its inactive state and leads to an increase in its deacetylase activity. Importantly, SIRT1 ISGylation promoted lung cancer progression and limited lung cancer cell sensitivity to DNA damage-based therapeutics in vivo and in vitro models. The levels of ISG15 mRNA and protein were significantly higher in lung cancer tissues than in adjacent normal tissues. Accordingly, elevated expression of SIRT1 and ISG15 was associated with poor prognosis in lung cancer patients, a finding that could be translated for lung cancer patient stratification and disease outcome evaluation. Taken together, our findings provide a mechanistic understanding of the regulatory effect of SIRT1 ISGylation on tumor progression and therapeutic efficacy in lung cancer.

## High ISG15 levels linked to poor prognosis in lung cancer patients

Interferon-stimulated gene 15 (ISG15) is known to influence tumor growth and severity. Young Joo Jeon at Chungnam National University College of Medicine, Daejon, South Korea, and co-workers found that ISG15 can conjugate to another protein, SIRT1, which also affects tumor growth and response to treatment. The research, conducted on human cells, mice, and human lung cancer tissues, revealed that when ISG15 conjugates to SIRT1, it boosts SIRT1’s activity, encouraging tumor growth and reducing the effectiveness of a chemotherapy drug. Additionally, high levels of SIRT1 and ISG15 in lung cancer tissues were linked to worse outcomes. The study suggests that focusing on understanding of the regulatory effect of ISG15 conjugation to SIRT1 could enhance cancer treatments. This summary was initially drafted using artificial intelligence, then revised and fact-checked by the author.

## Introduction

Interferon (IFN)-stimulated gene 15 (ISG15), the first identified ubiquitin-like protein (UBL), is highly expressed in many tumor types^1,2^. ISG15 functions in tumor development, tumor aggressiveness, cell self-renewal, and cell-to-cell communication in the tumor microenvironment^3–8^, suggesting that ISG15 is an active player in cancer pathogenesis rather than a passive observer. ISG15 exists in three distinct forms: unconjugated within the cell, conjugated to target proteins within the cell, and released into the extracellular space^9–11^. In a similar manner to ubiquitination, the conjugation of ISG15 to target proteins (ISGylation) is accomplished through a three-step enzymatic cascade involving E1 activating, E2 conjugating, and E3 ligase enzymes, and the reversal of ISGylation is accomplished by ubiquitin-specific protease 18 (USP18)^12–22^. Unlike the constitutive expression of some UBLs, ISG15 and the enzymes that catalyze ISGylation are robustly induced by IFNs, viral and bacterial infection, or genotoxic stresses^2,12,21,23–27^, indicating that ISGylation is a tightly fine-tuned process modulated by various physiological and pathophysiological perturbations. To comprehensively identify the targets of ISGylation, i.e., the “ISGylome”, numerous quantitative proteomics analyses have been performed^28–32^, and the results demonstrated that targets of ISGylation are associated with various signaling pathways in a cell- and tissue type-dependent manner. However, obtaining a mechanistic understanding of how ISGylation modulates cell proliferation and therapeutic efficacy in cancer is complicated due to the need to consider the specific tumor context. We previously reported that ΔNp63α ISGylation modulates epithelial tumor progression^23^. We found that ultraviolet-induced PCNA ISGylation is involved in the maintenance of genome stability^25^. Furthermore, we demonstrated that p53 ISGylation regulates its transactivity^33^. Our previous findings have therefore contributed to delineating the important roles of ISGylation in the control of cancer pathogenesis and the therapeutic response.

SIRT1 is a class III histone deacetylase that uses nicotinamide adenine dinucleotide (NAD^+^) as a cosubstrate for its enzymatic activity and deacetylates histone and nonhistone substrates, serving as a crucial link connecting multiple biological activities, including metabolism, inflammation, lifespan prolongation, and aging^34^. Importantly, SIRT1 is involved in tumor progression and the therapeutic response^35^. Furthermore, accumulating evidence indicates that SIRT1 activity is tightly modulated in response to multiple stresses^36^. Therefore, understanding the regulatory mechanism of SIRT1 in distinct contexts is highly important and could contribute to therapeutic interventions for cancer.

Here, we identified SIRT1 as a new target for ISGylation. SIRT1 ISGylation impairs the association of SIRT1 with its negative regulator, deleted in breast cancer 1 (DBC1), which unleashes SIRT1 from its inactive state and leads to an increase in its deacetylase activity. Accordingly, SIRT1 ISGylation promoted lung cancer progression and limited lung cancer cell sensitivity to DNA damage-based therapeutics in in vivo and in vitro models, and elevated expression of SIRT1 and ISG15 was associated with poor prognosis in lung cancer patients. Taken together, our findings provide a deep understanding of the molecular mechanism of SIRT1 ISGylation to determine its contribution to tumor progression and the response to therapy.

## Materials and methods

### Plasmids, shRNAs, and siRNAs

The Flag-ISG15, Flag-USP18, Myc-UbcH8, UBE1L, and Myc-DBC1 expression plasmids have been described previously^23,25^. Human SIRT1 cDNA and its deletion mutants were cloned into pcDNA4-HisMax (V864-20, Thermo Fisher Scientific, Waltham, MA, USA). The SIRT1-V5 (HsCD00871341) and AROS-V5 (HsCD00442690) expression plasmids were purchased from the Arizona State University (ASU) Biodesign Institute (Tempe, AZ, USA). Human AROS cDNA was cloned into pcDNA4-HisMax (V864-20, Thermo Fisher Scientific). Human ISG15 (M-004235-04-0010) and DBC1 (M-010427-01-0005) siGENOME SMARTpools and siGENOME nontargeting small interfering RNA (D-001206-14-20) were purchased from Dharmacon, a Horizon Discovery Group Co. (Cambridge, UK). sgControl-lentiCRISPRv2 (107402) was purchased from Addgene (Watertown, MA, USA).

### CRISPR‒Cas9-mediated genome editing

The SIRT1 KO cell line was generated by CRISPR‒Cas9-mediated genome editing. In brief, annealed oligonucleotides containing the gRNA target sequence (5′-CTCCGCAAGAGGCCGCGGAG-3′) were cloned into the lentiCRISPRv2 vector. The lentiCRISPRv2 vector containing the sgRNA against SIRT1 or sgControl was transfected into HEK293T cells along with the Gag-Pol (pCMV Δ8.91 R) and VSV-G (pMD-G) plasmids. Lentiviral particles were collected 48 h after transfection. A549 cells were transduced by incubation with the viral supernatant (1.5 ml), fresh medium (8.5 ml), and polybrene (7.5 μg/ml). After 24 h, the transduced cells were maintained in DMEM supplemented with 2 μg/ml puromycin. Typical colonies were picked from the plates using cloning cylinders, subcultured, and expanded. SIRT1 KO clonal cell lines were characterized using genomic DNA sequencing, immunocytochemistry, and immunoblotting with an anti-SIRT1 antibody.

### Lentivirus production and lentiviral transduction of SIRT1 KO A549 cells

To stably re-express SIRT1 in the SIRT1 KO clonal cell line, we used a lentiviral vector expressing the SIRT1 sgRNA-resistant SIRT1-V5 (HsCD00871341, ASU Biodesign Institute) or a lentiviral control vector (40125, Addgene). Lentiviruses were produced by transfecting a lentiviral vector and HIV packaging mix (LT002-01, GeneCopoeia, Rockville, MD, USA) into HEK293FT cells (R70007, Thermo Fisher Scientific) using Lipofectamine 2000 (11668027, Thermo Fisher Scientific). Lentiviral particles were collected 72 h after transfection. SIRT1 KO cells were then transduced with lentivirus in the presence of 10 μg/ml polybrene. After 36 h, the transduced cells were maintained in DMEM supplemented with 10 μg/ml blasticidin. Typical colonies were picked from the plates using cloning cylinders, subcultured, and expanded.

### Antibodies and chemicals

Antibodies against SIRT1 (5322, Sigma‒Aldrich, St. Louis, MO, USA; sc-15404, Santa Cruz, Dallas, TX, USA; or 07-131, Millipore, Temecula, CA, USA), ISG15 (15981-1-AP, Proteintech, Rosemont, IL, USA; or HPA004627, Atlas Antibodies, Bromma, Sweden), Ac-p53 (2525, Cell Signaling, Danvers, MA, USA), phospho-p53 (9284, Cell Signaling), p53 (sc-126, Santa Cruz), DBC1 (A300-434A, Bethyl Laboratories, Montgomery, TX, USA), cleaved PARP (9541, Cell Signaling), Xpress (R910-25, Thermo Fisher Scientific), Flag M2 (F1804, Sigma‒Aldrich), V5 (R960-25, Thermo Fisher Scientific), c-Myc (C3946, Sigma‒Aldrich; or sc-40, Santa Cruz), β-actin (sc-47778, Santa Cruz), and GAPDH (MA5-15738, Invitrogen, Waltham, MA, USA) were used. Anti-V5 agarose affinity gel was purchased from Sigma‒Aldrich (A7345). Doxorubicin (D1515), camptothecin (S1288), cisplatin (S1166), and recombinant human IFN-alpha A (alpha 2a) (11100-1) were purchased from Sigma‒Aldrich, Selleckchem (Houston, TX, USA), Selleckchem, and R&D Systems (Minneapolis, MN, USA), respectively.

### Cell culture and transfection

HEK293T, HEK293FT, C-33A, HeLa, A549, MCF7, Huh7 and HepG2 cells were maintained at 37 °C in a 5% CO_2_ atmosphere in DMEM (Welgene, Daegu, Korea) supplemented with 10% FBS (Gibco, Thermo Fisher, Waltham, MA, USA). H23 and T47D cells were cultured at 37 °C in a 5% CO_2_ atmosphere in RPMI 1640 (Welgene, Daegu, Korea) supplemented with 10% FBS. Transfection was performed using jetPRIME (114-75, Polyplus, Illkirch-Graffenstaden, FR), DharmaFECT1 (T-2001-03, Dharmacon) or Lipofectamine RNAiMAX (13778-150, Thermo Fisher Scientific).

### Immunoprecipitation and NTA pulldown

Cells were lysed in 50 mM Tris-HCl (pH 8) supplemented with 150 mM NaCl, 0.5% NP-40, 1 mM PMSF, and 1X protease inhibitor cocktail (11 697 498 001, Roche Applied Science, Basel, Switzerland) in the absence or presence of 0.25% SDS. The cell lysates were incubated with appropriate antibodies for 3 h at 4 °C, after which 20 μl of protein A/G agarose beads was added (sc-2003, Santa Cruz) and the mixture was incubated for an additional 2 h. For pulldown with NTA resin, cells were lysed in 50 mM Tris-HCl (pH 8) supplemented with 150 mM NaCl, 10 mM imidazole, 0.5% NP-40, 1 mM PMSF, and 1X protease inhibitor cocktail. The cell lysates were incubated with NTA resin (25215, Thermo Fisher Scientific) for 3 h at 4 °C. Harvested xenograft tumors were lysed in 50 mM Tris-HCl (pH 8) supplemented with 150 mM NaCl, 0.5% NP-40, 1 mM PMSF, and 1X protease inhibitor cocktail (11 697 498 001, Roche Applied Science) by using an ultrasonic homogenizer. The tumor lysates were incubated with appropriate antibodies for 3 h at 4 °C, after which 20 μl of protein A/G agarose beads was added (sc-2003, Santa Cruz) and the mixture was incubated for an additional 2 h.

### ISGylation sites mapping by mass spectrometry

V5-tagged SIRT1 WT was expressed in HEK293T cells in the presence of the ISG15-conjugating system and then purified via immunoprecipitation with an anti-V5 antibody. Proteins in the immunoprecipitates were separated by SDS–PAGE and visualized by staining with Coomassie blue R250. The Coomassie-stained gel was rinsed with water. The gel spot that was shifted upward from the position of main SIRT1 band was excised and chopped into smaller pieces. These gel pieces were destained with 100 mM ammonium bicarbonate/acetonitrile (1:1, v/v). In-gel digestion with trypsin was then performed as previously described^37^. LC–MS/MS analysis of the peptides extracted from the gel was performed on a Q Exactive mass spectrometer coupled to an UltiMate 3000 HPLC system (Thermo Fisher Scientific). In this LC‒MS system, the peptides were separated on an EASY-Spray column (15 cm × 75 µm I.D., C18, particle size of 2 µm, Thermo Fisher Scientific). Mobile phases A and B were 0.1% (v/v) formic acid and 0.1% (v/v) formic acid in 80% (v/v) acetonitrile, respectively. Peptides were eluted with a linear gradient from 2% to 40% buffer B over 55 min at a flow rate of 300 nL/min. The parameters for mass spectrometry were set as previously described^38^.

Mass spectra were analyzed with the SEQEST HT module in Proteome Discoverer 2.4 (Thermo Fisher Scientific) and searched against the human proteome database downloaded from UniProt (20,591 entries, Release 2023_01). The mass tolerance values were set to 10 ppm and 0.02 Da for the precursor and the fragment ions, respectively. The search parameters were as follows: full tryptic specificity with up to two missed cleavages and cysteine carbamidomethylation (+57.021 Da) as the static modification. The variable modifications included methionine oxidation (+15.995 Da) and lysine GlyGly modification (+114.043 Da). The false discovery rate (FDR) was set to 0.01 both at the peptide and at the peptide spectrum match (PSM) levels with the Percolator module. The resultant spectra were further filtered based on the XCorr scores (≥1.9, 2.2, and 3.75 for precursor charge states of +1, +2, and +3 or higher, respectively). Annotated MS/MS spectra were generated using the freely accessible web tool Interactive Peptide Spectrum Annotator (IPSA)^39^.

### SIRT1 activity assay

The deacetylase activity of SIRT1 was evaluated by using a SIRT1 Activity Assay Kit (ab156065, Abcam, Cambridge, UK) as recommended by the manufacturer. Briefly, cells were washed with cold PBS and lysed in 50 mM Tris-HCl (pH 8) supplemented with 150 mM NaCl and 0.5% NP-40 in the presence or absence of 10 mM imidazole. Cell lysates were subjected to pulldown with NTA resin (25215, Thermo Fisher Scientific) for 3 h at 4 °C or incubated with an anti-SIRT1 antibody for 3 h at 4 °C. Afterward, 20 μl of protein A/G agarose beads was added (sc-2003, Santa Cruz), and the mixture was incubated for an additional 2 h. The precipitates were incubated with Fluoro-Substrate Peptide Solution, NAD^+^, and SIRT1 assay buffer. The fluorescence intensity was subsequently measured using a microtiter plate fluorometer with an excitation wavelength of 350 nm and an emission wavelength of 450 nm.

### Cell growth and clonogenic assays

For the cell growth assay, cells (5 × 10^5^) were seeded and incubated in triplicate for 24 h. The cells were then treated with 1 μM doxorubicin for 24 h before harvesting. Viable cells were counted following trypan blue staining. For the clonogenic assay, cells were plated in 6-well plates (400 cells in 2 ml of medium per well). The medium was not changed during the experiment. After 13 days, the colonies were fixed, stained with crystal violet, and counted.

### TUNEL assay

Cells were cultured on glass coverslips. After incubation with 1 μM doxorubicin for the indicated times, the cells were fixed with 3.7% paraformaldehyde for 10 min and subjected to a terminal deoxynucleotidyl transferase-mediated dUTP-fluorescein nick end-labeling (TUNEL) assay with an in situ cell death detection kit following the manufacturer’s instructions (11684795910, Roche Applied Science).

### Animal studies

All animal experiments were approved by the Institutional Animal Care and Use Committee (IACUC) of Chungnam National University (Approval number: CNUH-020-A0020-1). Animal care was conducted according to institutional guidelines. SIRT1 KO A549 cells (7.5 × 10^6^) stably expressing empty vector, SIRT1 WT or SIRT1 KR were subcutaneously injected into the flanks of 6-week-old BALB/c nude mice. On the fourth day after injection, the mice were randomly allocated into different treatment groups (6 mice per group) and received intraperitoneal injections of PBS or doxorubicin (1.25 mg/kg) twice weekly for 5 weeks. The mice were monitored regularly for tumor growth. Tumor volumes were calculated as (a × a × b)/2, where a is the smallest diameter and b is the largest. At the end of the experiments, the mice were sacrificed, and the tumors were harvested.

### Lung cancer patients and tissue samples

Tumor and adjacent normal tissues obtained from adenocarcinoma lung cancer patients were provided by the Human Resources Bank of Chungnam National University Hospital (IRB number CNUH 2021-06-007). A cohort of lung cancer patients (*n* = 89, patients diagnosed between 2011 and 2012) was generated and represented patients diagnosed at Chonbuk National University Hospital. All cases were reviewed according to the WHO classification and the American Joint Committee Cancer Staging System. Information on clinicopathological factors was obtained by a review of medical records. All the samples were obtained with the approval of the Institutional Review Board of Chonbuk National University Hospital (IRB number CUH 2018-10-026-001). Analysis was performed in accordance with the tenets of the Declaration of Helsinki.

### Immunohistochemical staining and scoring

Dual-immunohistochemical staining for SIRT1 and ISG15 was performed using a Ventana BenchMark ULTRA system (Roche Korea Diagnostics Ltd., Seoul, Korea). Antigen retrieval was performed for 64 min using pH 9.0 CC1 antigen retrieval solution (Roche Korea Diagnostics Ltd.). The specimens were incubated with primary antibodies (anti-SIRT1 (sc-15404), 1:100, Santa Cruz; and anti-ISG15 (HPA004627), 1:100, Atlas Antibodies) for 90 min, and developed with an OptiView DAB IHC Detection Kit (Roche Korea Diagnostics Ltd.) for SIRT1 and with an UltraView Universal Alkaline Phosphatase Red Detection Kit (Roche Korea Diagnostics Ltd.) for ISG15. For quantification, the combined expression of SIRT1 and ISG15 (SIRT1/ISG15) and the individual expression of SIRT1 or ISG15 were assessed. The combined expression of SIRT1 and ISG15 (SIRT1/ISG15) and the individual expression of SIRT1 or ISG15 were scored by summing the staining intensity score (0; no staining, 1; weak staining, 2; intermediate staining, 3; strong staining) and the area score (0; 0%, 1; 1%, 2; 2–10%, 3; 11–33%, 4; 34–66%, 5; 67–100%)^40^. The final score was assessed by summing the scores from the two tissue microarray cores, resulting in a final immunohistochemical staining score ranging from zero to sixteen^41^.

### Analysis of lung cancer patients

The cutoff points for the scores for the combined expression of SIRT1 and ISG15 (SIRT1/ISG15) and the individual expression of SIRT1 or ISG15 were determined by receiver operating characteristic curve analysis. The cutoff points were determined as the points with the greatest area under the curve for predicting the death of lung cancer patients. Survival analysis was also performed to evaluate the overall survival of patients. The death of a lung cancer patient was considered a death event. Patients who were alive at last contact or who died from other causes were considered censored. To determine the relationships between clinicopathological factors and the prognostic impact of survival factors, Pearson’s chi-square test, univariate and multivariate Cox proportional hazards regression analyses, and Kaplan‒Meier survival analysis were performed using SPSS software (IBM, version 20.0, CA). *P* < 0.05 was considered to indicate statistical significance.

### Statistical analysis

The sample size and statistical significance of differences are described in the figure legends. All the quantitative data are presented as the mean ± SD or ±SEM of at least three independent experiments as determined by Student’s *t* test for between-group differences. *P* < 0.05 was considered to indicate statistical significance.

### Ethics approval

Lung cancer patient samples were obtained with the approval of the Institutional Review Board of Chonbuk National University Hospital (IRB number CUH 2018-10-026-001) or Chungnam National University Hospital (IRB number CNUH 2021-06-007). All animal experiments were approved by the Institutional Animal Care and Use Committee (IACUC) of Chungnam National University (Approval number: CNUH-020-A0020-1). Animal care was conducted according to institutional guidelines.

## Results

### SIRT1 is ISGylated

**Fig. 1:**
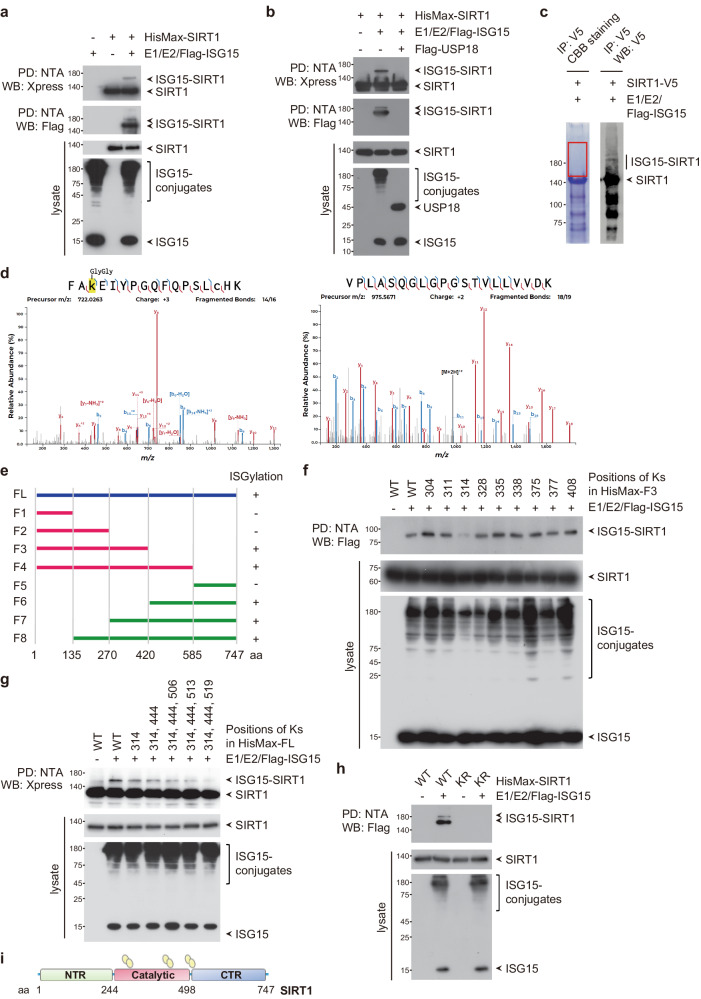
SIRT1 is a novel target for ISGylation. HisMax-SIRT1 or the ISG15-conjugating system (E1/E2/Flag-ISG15) were overexpressed alone or in combination in HEK293T cells without (**a**) and with (**b**) Flag-USP18 expression. Cell lysates were subjected to pulldown (PD) with NTA resin followed by Western blotting (WB) with an anti-Flag or anti-Xpress antibody. The lysates were also directly probed with the same antibodies. **c** Coomassie blue R250-stained gel showing enrichment of SIRT1-V5 immunoprecipitated from HEK293T cells. Coomassie blue R250-stained bands corresponding to ISGylated SIRT1-V5 were excised for MS/MS. Five percent of the SIRT1-V5- enriched immunoprecipitate was subjected to WB analysis with an anti-V5 antibody. **d** (Left) Annotated MS/MS spectrum of the peptide FAKEIYPGQFQPSLCHK from the SIRT1 protein. The Lys residue, highlighted in yellow, is at position 314 in the SIRT1 protein and is GlyGly modified (+114.042927 Da). The Cys residue of the peptide, indicated in lower case text, is carbamidomethylated (+57.021464 Da). (Right) Annotated MS/MS spectrum of the peptide VPLASQGLGPGSTVLLVVDK from the ISG15 protein. **e** Deletion mutants of HisMax-SIRT1 were generated. Whether each deletion was modified by ISG15 is indicated on the right. FL full-length. F fragment. HisMax-tagged F3 (**f**), FL SIRT1 and K-to-R mutants in various combinations (**g**) and SIRT1 WT or SIRT1 KR (**h**) were expressed in HEK293T cells along with the ISG15-conjugating system. Cell lysates were subjected to PD with NTA resin followed by WB with an anti-Xpress or anti-Flag antibody. **i** Domain structures of SIRT1. The ISGylation sites Lys314, Lys444, and Lys519 are denoted. NTR N-terminal regulatory domain, Catalytic catalytic core domain, CTR C-terminal regulatory domain.

To elucidate the mechanisms of action of ISG15 in cancer, we utilized a proteomic approach and analyzed proteins retrieved from HEK293T cells expressing the E1 activating enzyme UBE1L, the E2 conjugating enzyme UbcH8, and Flag-tagged ISG15 (collectively henceforth referred to as the ISG15-conjugating system) by immunoaffinity purification using anti-Flag antibody-immobilized resin. Among the putative targets for ISGylation (Supplementary Fig. 1), we selected SIRT1, as SIRT1 has been suggested to impact cancer pathogenesis. To verify whether SIRT1 is ISGylated, we coexpressed SIRT1 with the ISG15-conjugating system. Overexpression of SIRT1 with the ISG15-conjugating system led to the appearance of at least two ISGylated SIRT1 bands (Fig. 1a). Moreover, these bands disappeared upon coexpression of USP18, a major deISGylating enzyme (Fig. 1b), indicating that the bands correspond to ISGylated SIRT1.

To determine which lysine residues are critical for SIRT1 ISGylation, we performed mass spectrometry (MS) on SIRT1-V5 purified from HEK293T cells coexpressing SIRT1 and the ISG15-conjugating system. Coomassie blue R250-stained bands corresponding to ISGylated SIRT1 were excised for MS analysis (Fig. 1c). SIRT1 was identified with 72% sequence coverage (Supplementary Fig. 2). Among the identified peptides matched to SIRT1, two were found to be GlyGly modified (Fig. 1d, left and Supplementary Fig. 3) at Lys (K) 314 and K513 (highlighted in red in Supplementary Fig. 2). The GlyGly motif is from ISG15, as ISG15 was also identified in the same gel band (Fig. 1d, right and Supplementary Fig. 4). GlyGly modification results in the retention of the last two amino acid residues of ISG15 at SIRT1 after trypsin digestion. The GlyGly modified K314 peptide had more peptide spectral matches (PSMs) than did the corresponding K513 peptide. Using a label-free quantification (LFQ) approach, we compared the signal intensities of not only the GlyGly-modified K314 and K513 peptides but also the unmodified K314 and K513 peptides, assumed to have the amino acid sequences EIYPGQFQPSLCHK and LSEITEKPPR, respectively. The signal intensity of the GlyGly-modified K314 peptide was 5-fold greater than that of the GlyGly-modified K513 peptide, while the signal intensities of the peptides with unmodified K314 or K513 residues were similar (Supplementary Fig. 5), suggesting that K314 is the major potential site of ISGylation.

To further determine which lysine residues in SIRT1 are ISGylated, various deletion mutants of SIRT1 (termed fragment (F)1 to F8) were generated and expressed in HEK293T cells along with the ISG15-conjugating system. Full-length SIRT1 (FL; i.e., containing aa 1-747), F3 (containing aa 1-420), F4 (containing aa 1-585), F6 (containing aa 421-747), F7 (containing aa 271-747), and F8 (containing aa 136-747) but not F1 (containing aa 1-135), F2 (containing aa 1-270), or F5 (containing aa 586-747) were conjugated by ISG15, indicating that the deletion mutants containing aa 271-420 and aa 421-585 had ISGylation sites (Fig. 1e). In accordance with the results of our MS analysis (Fig. 1d), among the 9 Lys residues in the aa 271-420 sequence within F3, the substitution of Lys314 with arginine (Arg) but no other Lys (K)-to-Arg (R) substitutions noticeably reduced the ISGylation of F3 (Fig. 1f). Since the deletion mutants containing aa 421-585 also have ISGylation sites (Fig. 1e), we next replaced 10 Lys residues in the aa 420-585 sequence with Arg in full-length SIRT1 (FL) harboring the K314R substitution. Full-length SIRT1 harboring the K314R/K444R, K314R/K506R, K314R/K513R or K314R/K519R double substitution showed markedly diminished ISGylation (Supplementary Fig. 6). We, therefore, replaced Lys residues with Arg residues in full-length SIRT1 in various combinations. Notably, relative to the K314R/K444R/K513R triple substitution, the K314R/K444R/K519R triple substitution in SIRT1 (referred to herein as SIRT1 KR) almost completely abolished the ISGylation of SIRT1, even though K513 was identified as a putative site of ISGylation in the MS analysis (Fig. 1g, h). Considering the result of these analyses collectively, we suggest that Lys314 within the catalytic core domain is a major ISGylation site and that Lys444 within the catalytic core domain and Lys519 within the C-terminal extension are minor ISGylation sites in SIRT1 (Fig. 1i).

**Fig. 2:**
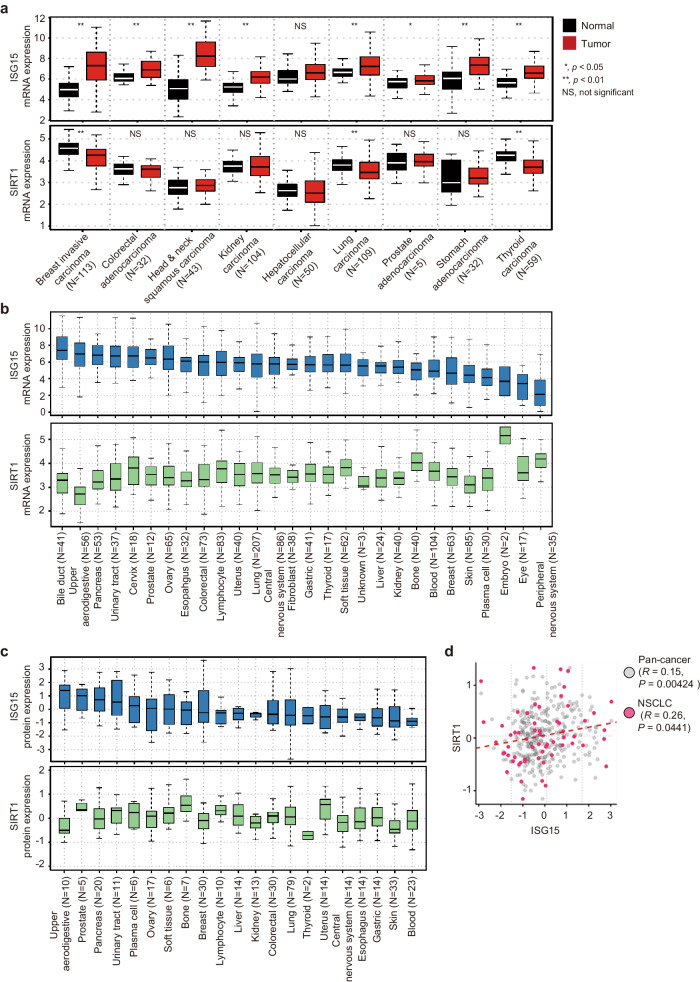
ISG15 is upregulated in several types of cancer. **a** mRNA expression levels of *ISG15* and *SIRT1* in paired tumor and adjacent normal tissues of nine tumor types in The Cancer Genome Atlas (TCGA) database. The TCGA RNA-seq data were obtained from the Genomic Data Commons (GDC), and log2-transformed transcripts per million (TPM) values are shown. **b** Boxplot showing the distribution of *ISG15* and *SIRT1* mRNA expression levels in 1404 cancer cell lines across 28 cancer types. mRNA expression values are presented as log2-transformed TPM values determined by RNA-seq using a pseudocount of 1. **c** Distribution of ISG15 and SIRT1 protein expression levels quantified by mass spectrometry in 375 cancer cell lines across 21 cancer types. Protein expression values are presented in a log2-transformed, normalized format. Proteomic and RNA-seq datasets of the cancer cell lines in the CCLE were obtained from the DepMap portal (https://depmap.org/portal/). **d** A scatter plot illustrating the relationship between ISG15 and SIRT1 protein expression across 375 cancer cell lines (depicted as gray dots) and 60 non-small cell lung carcinoma (NSCLC) cell lines (highlighted in red dots). The Pearson correlation coefficient (*R*) and the statistical significance of the correlation (*P*) were determined via a correlation test implemented in R software.

Given that the contributions of ISG15 and SIRT1 to cancer pathogenesis are complex and remain elusive, we analyzed the mRNA and protein expression levels of ISG15 and SIRT1 in The Cancer Genome Atlas (TCGA) and the Cancer Cell Line Encyclopedia (CCLE) database^42,43^. We first analyzed data from the TCGA database to compare the *ISG15* mRNA level among tumor tissues and adjacent normal tissues from patients with nine types of cancer. This analysis revealed that the *ISG15* mRNA level was significantly higher in all tumor tissues except for hepatocellular carcinoma tissues than in the corresponding adjacent normal tissues (Fig. 2a, upper). Our results are consistent with previous reports demonstrating the upregulation of ISG15 in several types of cancer^3,44–48^ and suggest that ISG15 is directly or indirectly involved in cancer development. However, the *SIRT1* mRNA level was not significantly different between the tumor and adjacent normal tissues in any cancers examined, except for invasive breast, lung, and thyroid carcinomas, in which the *SIRT1* mRNA level was significantly lower in the tumor tissues than in the adjacent normal tissues (Fig. 2a, lower). Further analyses of RNA sequencing (RNA-Seq) data from 1404 cancer cell lines across 28 cancer types and proteomic data from 375 cancer cell lines across 21 cancer types from the CCLE database revealed similar mRNAs and protein expression levels of ISG15 and SIRT1 in various cancer cell lines (Fig. 2b, c). Interestingly, the protein expression level of ISG15 showed a subtle yet significant positive correlation with the protein expression level of SIRT1 in the complete set of cancer types (*R* = 0.15, *P* = 0.0042) as well as in non-small cell lung carcinoma (NSCLC) (*R* = 0.26, *P* = 0.0441) (Fig. 2d).

**Fig. 3:**
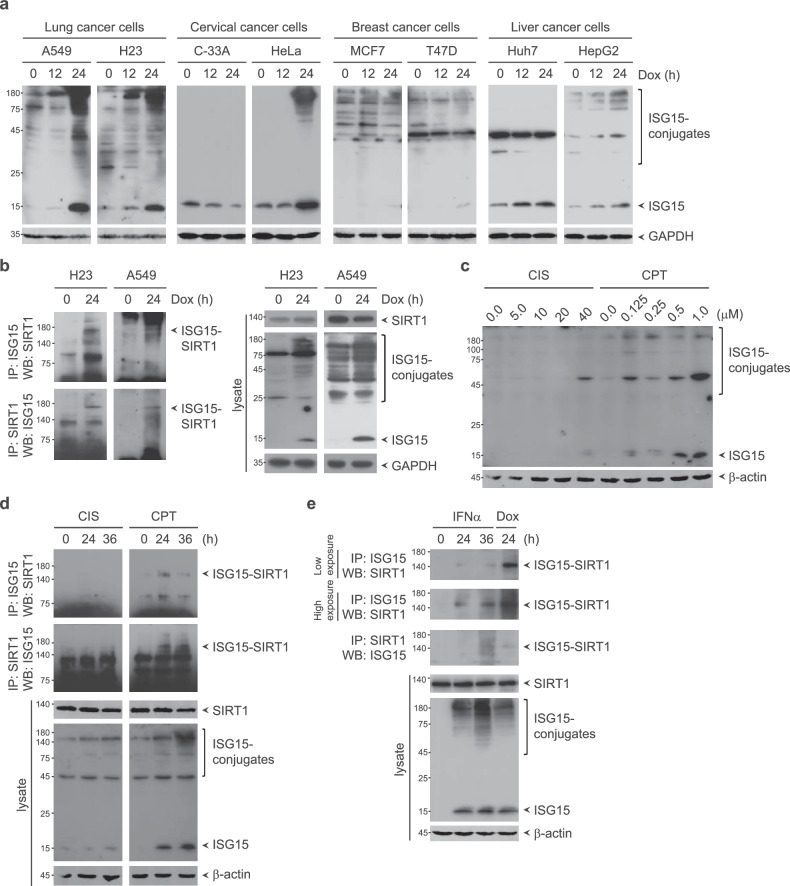
Induction of SIRT1 ISGylation in response to chemotherapeutic drugs. **a** A549, H23, C-33A, HeLa, MCF7, T47D, Huh7 and HepG2 cells were treated with 0.5 μM doxorubicin. Cell lysates were subjected to WB analysis with the indicated antibodies. **b** H23 and A549 cells incubated with doxorubicin were subjected to immunoprecipitation (IP) with an anti-ISG15 or anti-SIRT1 antibody followed by WB with an anti-SIRT1 or anti-ISG15 antibody, respectively. **c** A549 cells were incubated with increasing concentrations of cisplatin or camptothecin for 24 h. Cell lysates were subjected to WB analysis with the indicated antibodies. **d** A549 cells were treated with 40 μM cisplatin or 0.5 μM camptothecin. Cell lysates were subjected to IP with an anti-ISG15 or anti-SIRT1 antibody followed by WB with an anti-SIRT1 or anti-ISG15 antibody, respectively. **e** A549 cells were treated with 1000 U/ml IFNα or 0.5 μM doxorubicin. Cell lysates were subjected to IP with an anti-ISG15 or anti-SIRT1 antibody followed by WB with an anti-SIRT1 or anti-ISG15 antibody, respectively.

In contrast to the constitutive expression of some UBLs, the expression of ISG15 and the enzymes that catalyze ISGylation is strongly induced by physiological and pathophysiological perturbations. Moreover, we previously reported that doxorubicin, a DNA-damaging chemotherapeutic, robustly induces ISG15 expression and the formation of protein ISGylation conjugates^23,25,33^. Therefore, we determined whether doxorubicin induces ISG15 expression and the formation of protein ISGylation conjugates in cell lines derived from lung, cervical, breast or liver cancer. Significant induction of both ISG15 expression and protein ISGylation conjugates formation in the presence of doxorubicin was observed in A549 and H23 cells derived from lung cancer and in HeLa cells derived from cervical cancer (Fig. 3a). However, doxorubicin treatment had little or no effect on the induction of ISG15 expression and protein ISGylation conjugates formation in C33A cells derived from cervical cancer. Liver cancer-derived HepG2 cells but not Huh7 cells exhibited moderate induction of both ISG15 expression and protein ISGylation conjugates formation. MCF7 and T47D cells derived from breast cancer showed weak induction of ISG15 expression and protein ISGylation conjugates formation, even though protein ISGylation conjugates were present even in the absence of doxorubicin. We further investigated whether doxorubicin induces SIRT1 ISGylation in cell lines derived from lung, cervical, breast or liver cancer. Doxorubicin induced ISGylation of endogenous SIRT1 in both A549 and H23 cells derived from lung cancer, in which it was robustly able to induce ISG15 expression and protein ISGylation conjugates formation (Fig. 3b). Furthermore, HeLa cells derived from cervical cancer and MCF7 cells derived from breast cancer showed significant evidence of SIRT1 ISGylation upon doxorubicin treatment (Supplementary Fig. 7), suggesting that SIRT1 is a bona fide target for ISGylation in a subset of cancer cells. Intriguingly, SIRT1 has been reported to play an important role in lung cancer development and chemoresistance^49^. Therefore, these findings prompted us to focus on lung cancer to explore the role of SIRT1 ISGylation in cancer pathogenesis. However, further studies might be required to expand the understanding of SIRT1 ISGylation in various cancers.

DNA-damaging chemotherapeutics reportedly upregulate ISGylation^23^. For example, camptothecin increases the levels of free ISG15 and its conjugates in a dose- and time-dependent manner^50^. We therefore examined whether other chemotherapeutic drugs, i.e., camptothecin and cisplatin, induce ISG15 expression and protein ISGylation conjugates formation. In A549 cells, camptothecin treatment induced ISG15 expression and protein ISGylation conjugates formation, whereas cisplatin treatment had little or no effect on the induction of ISG15 expression and protein ISGylation conjugates formation (Fig. 3c, d). Accordingly, camptothecin but not cisplatin induced the ISGylation of endogenous SIRT1 (Fig. 3d).

Type I IFNs activate the expression of hundreds of ISGs and regulate tumorigenesis through both tumor cell-intrinsic and tumor cell-extrinsic mechanisms, leading to tumor progression or tumor suppression in a context-dependent manner^8,51,52^. ISG15 and the enzymes that catalyze ISGylation are robustly induced by type I IFNs. Hypoxia-inducible factor-1α (HIF-1α) has been reported to be ISGylated upon IFN treatment, which reduces the transcriptional activity of HIF-1α and subsequently affects tumor growth^53^. The type I IFN-mediated ISG–ISGylation network in the tumor microenvironment of breast cancer synergistically induces the expression of chemokine receptor ligands and attracts cytotoxic T cells, thereby orchestrating the establishment of a tumor-suppressive microenvironment^8^. In contrast, tumor cell resistance to type I IFNs has been demonstrated^54–57^. Type I IFN-induced filamin B ISGylation acts as a negative feedback regulatory gate for the desensitization of IFN-induced JNK signaling and apoptosis^58^. Parkin ISGylation induced by type I IFN promotes the ubiquitin E3 ligase activity of parkin, thereby increasing the protective effect of parkin against IFN-induced cell death^59^, suggesting the double-edged roles of type I IFN-induced protein ISGylation in controlling tumor progression or tumor suppression. We investigated whether SIRT1 can be ISGylated upon type I IFN treatment. SIRT1 was weakly ISGylated upon IFN treatment compared with doxorubicin treatment, even though the IFN and doxorubicin induced ISG15 expression and protein ISGylation conjugates formation to comparable degrees (Fig. 3e). A particular E2 conjugating or E3 ligase enzyme for SIRT1 ISGylation might be differentially activated by different stimuli, suggesting that complex regulatory mechanisms may underlie ISGylation in a manner dependent on the biological context.

### ISGylation enhances the deacetylase activity of SIRT1

**Fig. 4:**
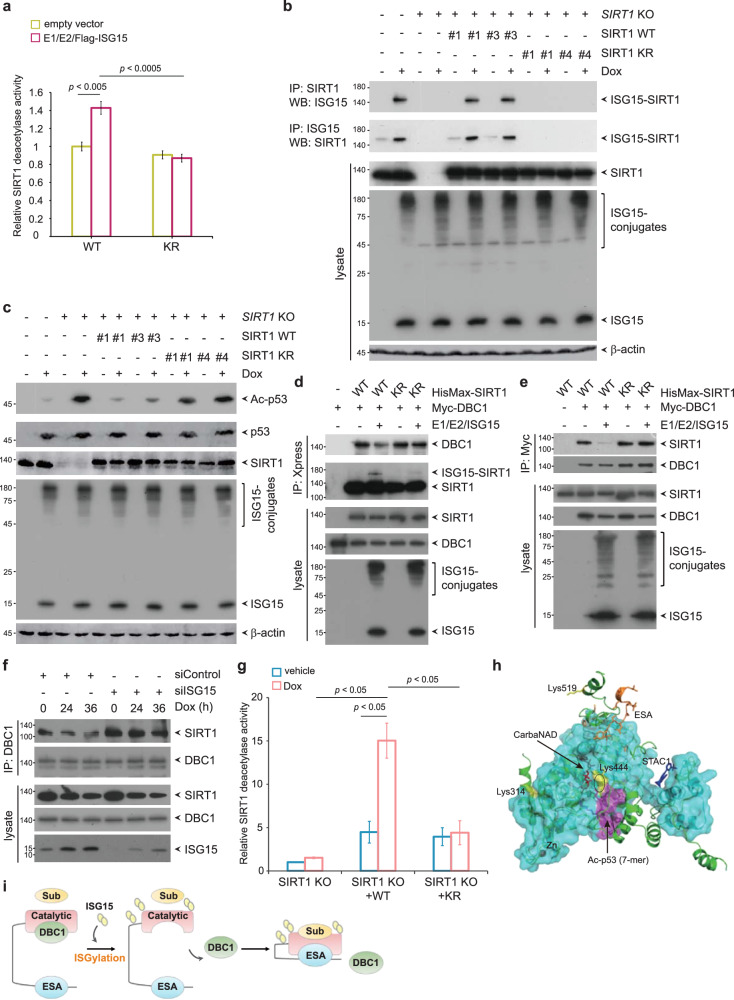
ISGylation of SIRT1 promotes its deacetylase activity. **a** SIRT1 WT and SIRT1 KR in the presence or absence of the ISG15-conjugating system were purified via PD with NTA resin from HEK293T cells and assayed for deacetylase activity using a fluorometric assay system. The bars indicate the mean ± SD of three independent experiments. **b** Of the many clones (Supplementary Fig. 8e), SIRT1 KO clone #1, SIRT1 KO + WT clones #1 and #3, SIRT1 KO + KR clones #1 and #4, and Control cells were chosen and treated with 1 μM doxorubicin for 24 h. Cell lysates were subjected to IP with an anti-SIRT1 or anti-ISG15 antibody followed by WB with an anti-ISG15 or anti-SIRT1 antibody, respectively. **c** Lysates of the cells treated as described in (b) were subjected to WB analysis with the indicated antibodies. **d**, **e** HisMax-tagged SIRT1 WT or SIRT1 KR was transiently coexpressed with Myc-tagged DBC1 with or without the ISG15-conjugating system. Cell lysates were subjected to IP with an anti-Xpress antibody (**d**) or anti-Myc antibody (**e**) followed by WB with an anti-Myc or anti-Xpress antibody, respectively. **f** A549 cells were transfected with siControl or siISG15 and incubated with 0.5 μM doxorubicin for the indicated times. Cell lysates were subjected to IP with an anti-DBC1 antibody followed by WB with an anti-SIRT1 antibody. **g** SIRT1 was purified via IP with an anti-SIRT1 antibody from SIRT1 KO cells, SIRT1 WT-complemented SIRT1 KO cells and SIRT1 KR-complemented SIRT1 KO cells with or without doxorubicin treatment and assayed for deacetylase activity using a fluorometric assay system. The bars indicate the mean ± SD of three independent experiments. **h** ISGylation sites in the 3D structure of SIRT1. The structure of mini-hSIRT1 (PDB ID: 4ZZJ) is shown as a ribbon diagram with a transparent cyan surface. The green ribbon represents a model of full-length human SIRT1 generated via AlphaFold but with the structurally inaccurate long loops removed for clarity. The ISGylated lysine residues are depicted as yellow sticks and labeled. The bound cosubstrate analogs carbanicotinamide adenine dinucleotide (CarbaNAD) and sirtuin-activating compound (STAC1) are shown as red and blue sticks, respectively. The enzymatic product, the Ac-p53 peptide, is shown as a magenta surface, and the acetylated lysine residue is indicated by an oval. The ESA region in the AlphaFold model is shown in orange. Note that the N-terminal STAC1 binding domain shows different conformations between the experimental and AlphaFold models. **i** A schematic diagram of the regulation of SIRT1 catalytic activity by ISGylation. SIRT1 ISGylation impairs the association of SIRT1 with DBC1, which unleashes SIRT1 from its inactive state and leads to an increase in its deacetylase activity. Furthermore, SIRT1 ISGylation might enhance the intramolecular interaction between the catalytic core domain and the ESA region or increase substrate accessibility.

To assess the molecular and functional consequences of ISGylation on SIRT1, we examined whether ISGylation affects the deacetylase activity of SIRT1. SIRT1 WT or SIRT1 KR was expressed in HEK293T cells in the absence or presence of the ISG15-conjugating system and was purified via pulldown with NTA resin (Supplementary Fig. 8a). The purified proteins were then subjected to an in vitro fluorometric assay to measure deacetylase activity. SIRT1 WT showed a significant increase in deacetylase activity in the presence of the ISG15-conjugating system compared to in the absence of the ISG15-conjugating system (Fig. 4a). However, SIRT1 KR showed no increase in deacetylase activity in the presence of the ISG15-conjugating system, suggesting that ISGylation substantially increases the deacetylase activity of SIRT1. To further improve the understanding of this phenomenon, we used CRISPR‒Cas9 genome editing to ablate SIRT1 and validated the expression of SIRT1 in independent SIRT1 knockout (SIRT1 KO) A549 clonal cell lines via immunoblotting with an anti-SIRT1 antibody (Supplementary Fig. 8b). Among the SIRT1 KO clonal cell lines, we selected clone #2 and further verified the ablation of SIRT1 using immunocytochemistry and genomic DNA sequencing analysis (Supplementary Fig. 8c, d). We also established sgControl- and lentiviral control vector-expressing A549 clonal cells (henceforth referred to as Control cells), two lentiviral control vector-expressing SIRT1 KO cell lines, four SIRT1 sgRNA-resistant SIRT1 WT-complemented SIRT1 KO clonal cell lines, and four SIRT1 sgRNA-resistant SIRT1 KR-complemented SIRT1 KO clonal cell lines and validated the expression of SIRT1 in these cell lines (Supplementary Fig. 8e). We selected clone #1 among the two lentiviral control vector-expressing SIRT1 KO cell lines, clones #1 and #3 among the four SIRT1 WT-complemented SIRT1 KO cell lines, clones #1 and #4 among the four SIRT1 KR-complemented SIRT1 KO cell lines, and Control cells and examined the protein levels of SIRT1, ISG15 and ISGylation conjugates in the absence or presence of doxorubicin. Overall, the protein levels of SIRT1, ISG15 and protein ISGylation conjugates were comparable among the clonal cell lines (Fig. 4b). Importantly, doxorubicin significantly induced SIRT1 ISGylation in SIRT1 WT-complemented SIRT1 KO cells and Control cells but not in SIRT1 KR-complemented SIRT1 KO cells (Fig. 4b). Subsequently, clone #1 among the control vector-expressing SIRT1 KO cell lines, clone #1 among the SIRT1 WT-complemented cell lines, and clone #1 among the SIRT1 KR-complemented cell lines were selected and referred to as SIRT1 KO, SIRT1 WT-complemented SIRT1 KO, and SIRT1 KR-complemented SIRT1 KO cells, respectively. We expressed p53 in SIRT1 KO, SIRT1 WT-complemented and SIRT1 KR-complemented SIRT1 KO cells in the absence or presence of the ISG15-conjugating system. Consistent with the role of ISGylation in the deacetylase activity of SIRT1, deacetylation of p53 by SIRT1 WT was potentiated compared to that by SIRT1 KR in the presence of the ISG15-conjugating system (Supplementary Fig. 8f, g). In accordance with this finding, acetylation of endogenous p53 in the presence of doxorubicin was markedly increased in SIRT1 KR-complemented SIRT1 KO cells to a level approximately equal to that in SIRT1 KO cells, while p53 acetylation in the presence of doxorubicin was only marginally increased in SIRT1 WT-complemented SIRT1 KO cells and Control cells (Fig. 4c).

Based on the substantial role of ISGylation in the deacetylase activity of SIRT1, we further examined the underlying mechanisms in more detail. DBC1, a tumor suppressor originally identified as a protein not expressed in breast cancer cells, binds to SIRT1 and inhibits its enzymatic activity^60,61^. We therefore hypothesized that SIRT1 ISGylation might influence the interaction between SIRT1 and its negative regulator DBC1. DBC1 and SIRT1 WT or DBC1 and SIRT1 KR were coexpressed in HEK293T cells in the absence or presence of the ISG15-conjugating system and were reciprocally coimmunoprecipitated using antibodies against Xpress or Myc. Coexpression of the ISG15-conjugating system strongly impaired the interaction of DBC1 with SIRT1 WT but not with SIRT1 KR (Fig. 4d, e), indicating that SIRT1 ISGylation markedly promotes the dissociation of SIRT1 from DBC1. To investigate whether doxorubicin-induced SIRT1 ISGylation influences the physical interaction and functional connection between SIRT1 and DBC1, we coimmunoprecipitated endogenous SIRT1 and DBC1 from *ISG15* small interfering RNA (siRNA) (siISG15)-expressing or siControl-expressing cells with and without doxorubicin treatment. Importantly, the interaction of SIRT1 with DBC1 was attenuated following doxorubicin treatment in siControl-expressing cells (Fig. 4f). However, ISG15 depletion led to the persistent association of SIRT1 with DBC1 following doxorubicin treatment, suggesting that SIRT1 ISGylation induced by doxorubicin negatively regulates the association of SIRT1 with DBC1. We further examined whether doxorubicin-induced SIRT1 ISGylation affects the deacetylase activity of SIRT1. SIRT1 was purified from SIRT1 WT- and SIRT1 KR-complemented SIRT1 KO cells with or without doxorubicin treatment via immunoprecipitation with an antibody against SIRT1. SIRT1 WT but not SIRT1 KR was ISGylated upon doxorubicin treatment (Supplementary Fig. 8h). The purified proteins were then subjected to an in vitro fluorometric assay to measure their deacetylase activity. As expected, SIRT1 WT showed a significant increase in its deacetylase activity in the presence of doxorubicin compared with in the absence of doxorubicin (Fig. 4g). However, SIRT1 KR showed no increase in deacetylase activity in the presence of doxorubicin, suggesting that doxorubicin-induced SIRT1 ISGylation significantly increases the deacetylase activity of SIRT1. Active regulator of SIRT1 (AROS), also known as ribosomal protein S19 binding protein 1 (RPS19BP1), interacts with SIRT1 and amplifies the deacetylation capacity of SIRT1^62^. We therefore examined whether SIRT1 ISGylation regulates the physical interaction between SIRT1 and AROS. AROS and SIRT1 WT or AROS and SIRT1 KR were coexpressed in HEK293T cells in the absence or presence of the ISG15-conjugating system and were reciprocally coimmunoprecipitated using antibodies directed against V5 or Xpress. SIRT1 ISGylation did not influence the interaction between SIRT1 and AROS (Supplementary Fig. 9).

Analysis of known crystal structures to search for common features of the sequences surrounding the identified ISGylation target Lys residues has suggested that ISGylation occurs at sites associated with protein‒protein interactions, in dimerization domains or in enzyme active sites^32^. Although the 3D structure of full-length mammalian SIRT1 is not yet available, we were able to map the ISGylation sites in the human SIRT1 model based on the partial structure of SIRT1 in complex with a substrate peptide and NAD^+^ analog (Fig. 4h)^63^. As described above, Lys314 and 444 are in the catalytic domain of SIRT1. In particular, Lys444 is located near the catalytic region where the cosubstrate NAD^+^ and the substrate p53 bind. Lys519 is located in the concealed region outside the catalytic domain toward the C-terminal domain in the experimental structure. Based on the model generated via AlphaFold^64^, we estimated the position of the Lys519 residue, which is relatively close to the essential for SIRT1 activity (ESA) region located in the C-terminus of SIRT1. The intramolecular interaction between the catalytic core domain and the ESA region is essential for the catalytic activity of SIRT1^65^. Therefore, SIRT1 ISGylation might increase the accessibility of substrates or enhance the interaction between the catalytic core domain and the ESA region. Taken together, our findings suggest that SIRT1 ISGylation results in dissociation of the catalytic core domain from DBC1, which unleashes SIRT1 from its inactive state and leads to an increase in its deacetylase activity (Fig. 4i).

### SIRT1 ISGylation is required for lung cancer progression and limits sensitivity to DNA damage-based therapeutics

**Fig. 5:**
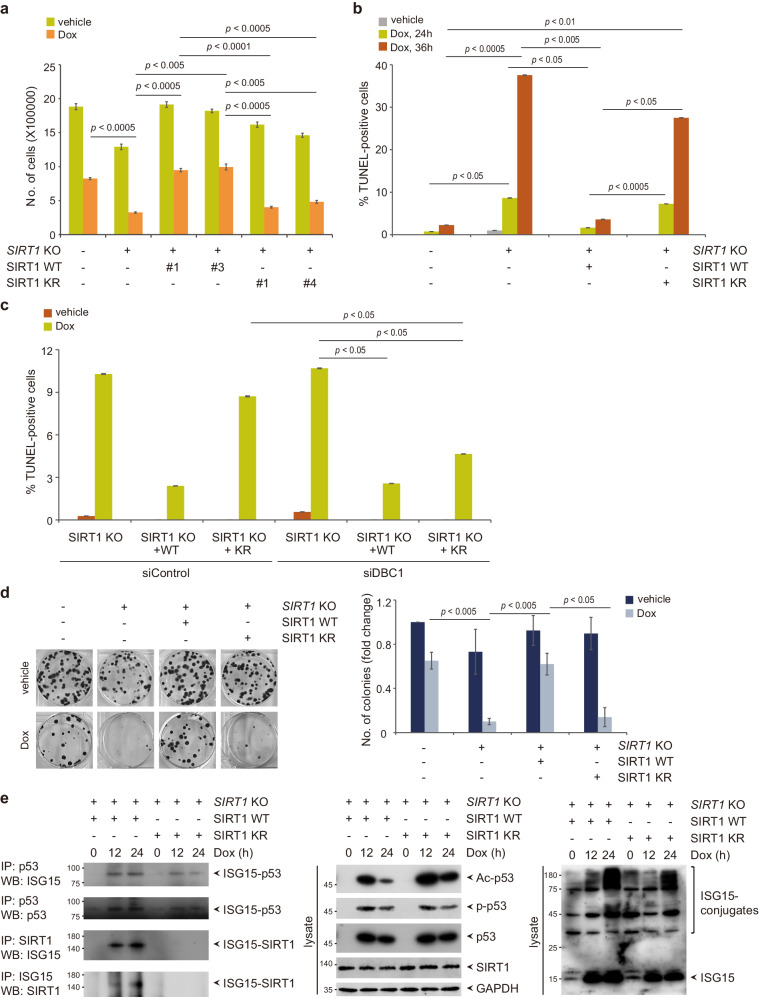
SIRT1 ISGylation plays an essential role in lung cancer cells proliferation and the response of lung cancer cells to DNA-damaging therapeutics. **a** SIRT1 KO clone #1, SIRT1 KO + WT clones #1 and #3, SIRT1 KO + KR clones #1 and #4, and Control cells were treated with 1 μM doxorubicin for 24 h. Viable cells were counted using trypan blue exclusion. The bars indicate the mean ± SD of three independent experiments. **b** SIRT1 KO cells, SIRT1 KO cells complemented with SIRT1 WT or SIRT1 KR, and Control cells were incubated with 1 μM doxorubicin for the indicated times. The cells were then subjected to a TUNEL assay. The number of TUNEL-positive cells was determined and is expressed as a percentage of the total number of cells. The bars indicate the mean ± SD of three independent experiments. **c** SIRT1 KO cells and SIRT1 WT- or SIRT1 KR-complemented SIRT1 KO cells were transfected with siControl or siDBC1 and incubated with 1 μM doxorubicin for 24 h. The cells were then subjected to a TUNEL assay. The number of TUNEL-positive cells was determined and is expressed as a percentage of the total number of cells. The bars indicate the mean ± SD of three independent experiments. **d** SIRT1 KO cells, SIRT1 KO cells complemented with SIRT1 WT or SIRT1 KR, and Control cells were incubated with or without 0.1 μM doxorubicin for 13 days. Colonies were stained with crystal violet and counted. The bars indicate the mean ± SD of three independent experiments. **e** SIRT1 WT-complemented and SIRT1 KR-complemented SIRT1 KO cells were treated with 1 μM doxorubicin for the indicated times. Cell lysates were subjected to IP with an anti-SIRT1 or anti-ISG15 antibody followed by WB with an anti-ISG15 or anti-SIRT1 antibody, respectively, or subjected to IP with an anti-p53 antibody followed by WB with an anti-ISG15 or anti-p53 antibody. Cell lysates were also subjected to WB analysis with the indicated antibodies.

To determine the potential effects of SIRT1 ISGylation on lung cancer progression and therapeutic efficacy, we first examined the impact of SIRT1 ISGylation on lung cancer cell proliferation. We selected clone #1 among the two lentiviral control vector-expressing SIRT1 KO cell lines, clones #1 and #3 among the four SIRT1 WT-complemented SIRT1 KO cell lines, clones #1 and #4 among the four SIRT1 KR-complemented SIRT1 KO cell lines, and Control cells (see Fig. 4b). Doxorubicin significantly induced SIRT1 ISGylation in SIRT1 WT-complemented SIRT1 KO and Control cells but not in SIRT1 KR-complemented SIRT1 KO cells (Supplementary Fig. 10a). Compared with Control cells, SIRT1 KO cells exhibited significant attenuation of cell growth (Fig. 5a). Compared with SIRT1 ablation, complementation of SIRT1 KO cells with either SIRT1 WT or SIRT1 KR facilitated cell growth. Following doxorubicin treatment, compared to SIRT1 ablation, SIRT1 WT complementation significantly attenuated the reduction in cell growth, whereas SIRT1 KR complementation led to only marginal attenuation of the doxorubicin-mediated reduction in cell growth, suggesting the important role of SIRT1 ISGylation in negatively regulating doxorubicin-mediated inhibition of lung cancer cell proliferation. We next investigated whether SIRT1 ISGylation could be implicated in doxorubicin-induced apoptosis. Doxorubicin treatment of SIRT1 KO cells increased not only the cleavage of poly (ADP-ribose) polymerase (PARP) but also the number of TUNEL-positive apoptotic cells (Fig. 5b, Supplementary Fig. 10b, and Supplementary Fig. 11). Importantly, SIRT1 WT complementation but not SIRT1 KR complementation in SIRT1 KO cells markedly attenuated doxorubicin-induced apoptosis, suggesting that SIRT1 ISGylation negatively regulates doxorubicin-induced apoptosis. Notably, the facilitation of apoptosis in SIRT1 KR-complemented cells upon doxorubicin treatment was mitigated following depletion of DBC1 (Fig. 5c and Supplementary Fig. 12), substantiating the importance of ISGylation-mediated regulation of the association between SIRT1 and DBC1 in controlling doxorubicin-mediated apoptosis. Moreover, compared to SIRT1 ablation, either SIRT1 WT or SIRT1 KR complementation in SIRT1 KO cells led to an increase in the colony formation ability to a level comparable to that of Control cells (Fig. 5d), suggesting the key role of SIRT1 in clonogenic growth. Interestingly, SIRT1 depletion led to a marked decrease in clonogenic growth following doxorubicin treatment. However, compared to SIRT1 KR complementation, SIRT1 WT complementation in SIRT1 KO cells markedly attenuated the doxorubicin-mediated inhibition of clonogenic growth, suggesting that SIRT1 ISGylation is required for lung cancer cell proliferation and survival.

It has been reported that p53 is ISGylated upon DNA damage^33^. Upon DNA damage, acetylation and phosphorylation of p53 precede its ISGylation to induce the expression of the ISG15-conjugating system as well as other downstream targets, which leads to further increases in the phosphorylation and acetylation of p53, thereby leading to the suppression of cell growth and tumor development in colorectal carcinoma. To determine whether SIRT1 ISGylation influences p53 ISGylation, SIRT1 WT-complemented and SIRT1 KR-complemented SIRT1 KO cells were treated with doxorubicin for the indicated times. The level of p53 ISGylation increased for 12 h after doxorubicin treatment and remained elevated in SIRT1 WT-complemented and SIRT1 KR-complemented SIRT1 KO cells, even though compared with SIRT1 KR complementation, SIRT1 WT complementation slightly potentiated p53 ISGylation (Fig. 5e). The level of SIRT1 ISGylation in SIRT1 WT-complemented but not in SIRT1 KR-complemented SIRT1 KO cells increased for 12 h after doxorubicin treatment and continued to increase further. SIRT1 ISGylation had little or no effect on p53 phosphorylation. Importantly, immunoblot analysis revealed that in SIRT1 KR-complemented SIRT1 KO cells, the level of p53 acetylation markedly increased for 12 h after doxorubicin treatment and remained elevated or began to subtly decrease thereafter. In contrast, in SIRT1 WT-complemented cells, the level of p53 acetylation increased for 12 h after doxorubicin treatment and drastically decreased thereafter, concomitant with a further increase in SIRT1 ISGylation. These results suggest that SIRT1 ISGylation is upregulated with a further increase in response to doxorubicin and thereby decreases p53 acetylation, with stronger effects than the p53 ISGylation-mediated increase in p53 acetylation, which leads to limitation of cancer cell sensitivity to doxorubicin. However, further studies to expand the understanding of not only the crosstalk network between SIRT1 ISGylation and p53 ISGylation but also the differential consequences of these processes depending on the tumor context are likely worthwhile.

**Fig. 6:**
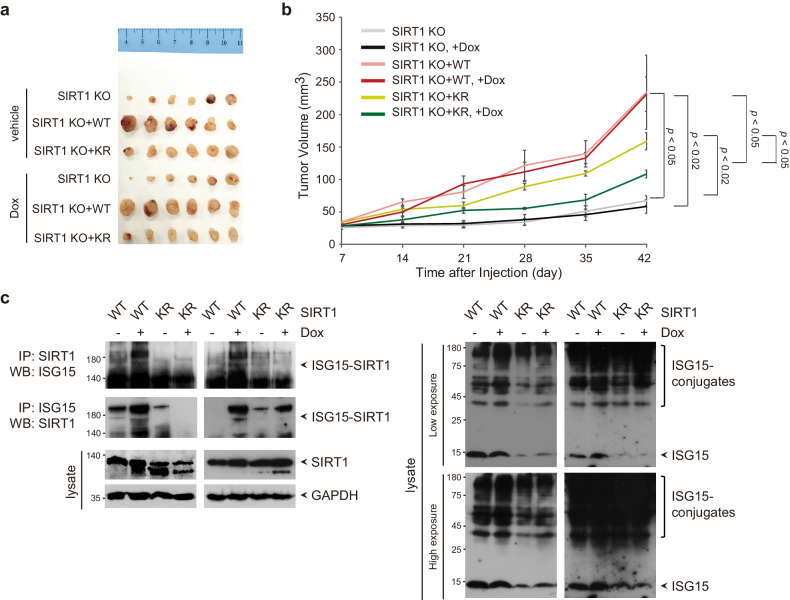
SIRT1 ISGylation limits tumor responses to doxorubicin. **a**, **b** SIRT1 KO cells and SIRT1 KO cells complemented with SIRT1 WT or SIRT1 KR were injected into BALB/c nude mice, and the mice were monitored weekly for tumor growth. The animals were subjected to doxorubicin treatment 4 days after injection, as described in the Methods section. The mice were sacrificed, and tumors were harvested (**a**). The bars indicate the means ± SEMs. *n* = 6 mice per treatment group (**b**). **c** Tumors formed as described in (**a**) were lysed and subjected to IP with an anti-SIRT1 or anti-ISG15 antibody followed by WB with an anti-ISG15 or anti-SIRT1 antibody, respectively. The lysates were also directly probed with the indicated antibodies.

Based on the above findings that SIRT1 ISGylation promotes cell proliferation and suppresses apoptosis, it is reasonable to postulate that SIRT1 ISGylation plays an important role in lung cancer progression and sensitivity to DNA damage-based therapeutics. To establish the functional role of SIRT1 ISGylation in lung cancer progression and the response to DNA-damaging chemotherapeutics in vivo, we engrafted SIRT1 KO, SIRT1 WT-complemented and SIRT1 KR-complemented cells into BALB/c nude mice. Compared with SIRT1 depletion, SIRT1 WT and SIRT1 KR complementation significantly increased tumor growth in BALB/c nude mice (Fig. 6a, b), substantiating the importance of SIRT1 in driving lung cancer progression. The size of tumors formed from SIRT1 WT-complemented cells in the absence of doxorubicin was greater than that of tumors formed from SIRT1 KR-complemented cells, possibly due to SIRT1 ISGylation resulting from biological events in vivo during tumor development. Intriguingly, SIRT1 WT complementation facilitated tumor growth and rendered tumors resistant to doxorubicin, whereas SIRT1 KR complementation attenuated tumor growth and sensitized tumors to doxorubicin. Taken together, our results suggest that SIRT1 ISGylation promotes lung cancer progression and limits sensitivity to doxorubicin. We sought to validate the ISGylation of SIRT1 in tumors. In accordance with our in vitro study, tumors formed from SIRT1 WT-complemented cells displayed an increase in SIRT1 ISGylation in the presence of doxorubicin compared with that in tumors formed from SIRT1 KR-complemented cells (Fig. 6c).

### SIRT1 ISGylation is correlated with poor prognosis in human lung cancer

**Fig. 7:**
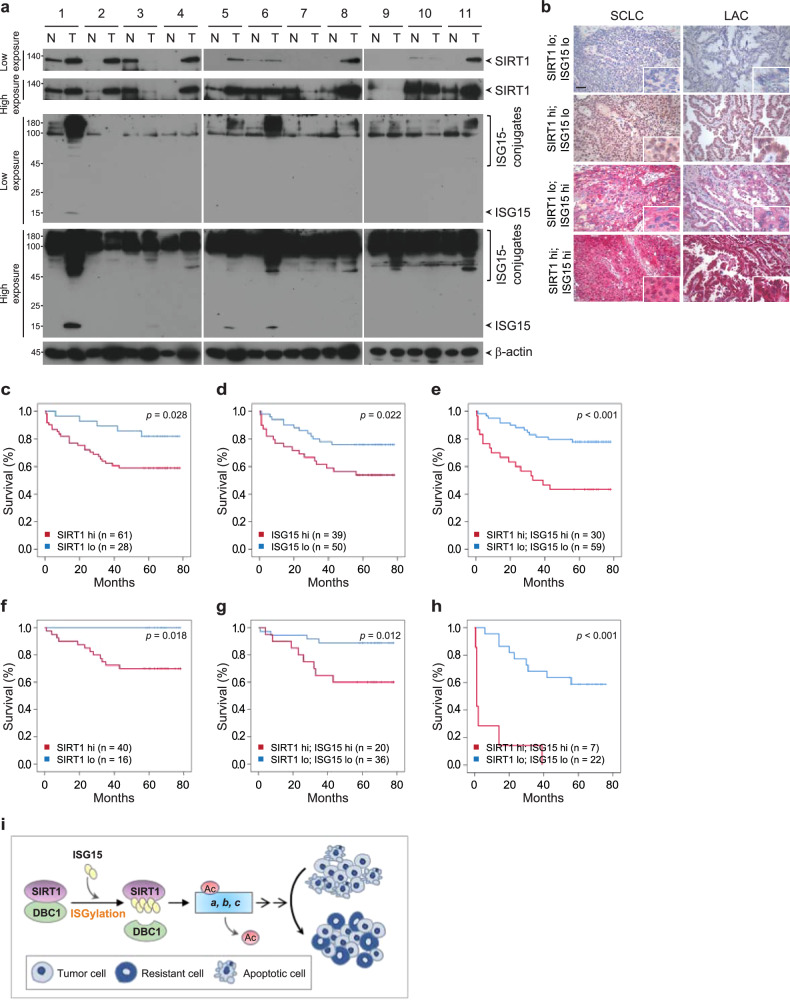
SIRT1 ISGylation serves as a prognostic marker in human NSCLCs. **a** Tumor tissues and the paired adjacent normal tissues derived from 11 lung adenocarcinoma patients were lysed and subjected to WB with the indicated antibodies. **b** Representative immunostaining of squamous cell lung carcinomas (SCLCs) and lung adenocarcinomas (LACs) depicting high and low levels of SIRT1 and ISG15 expression. SIRT1 staining appears brown, and ISG15 staining appears red. Original magnification, 400x. Scale bar = 50 μm. Kaplan–Meier curves comparing the OS of lung cancer patients with tumors expressing high or low levels of SIRT1 (*P* = 0.028) (**c**), high or low levels of ISG15 (*P* = 0.022) (**d**), or high or low levels of SIRT1 combined with high or low levels of ISG15 (*P* < 0.001) (**e**). **f**, **g** Kaplan–Meier curves comparing the survival of squamous cell lung carcinoma patients with tumors expressing high or low levels of SIRT1 (*P* = 0.018) (**f**) and high or low levels of SIRT1 combined with high or low levels of ISG15 (*P* = 0.012) (**g**). **h** Kaplan‒Meier curve comparing the survival of adenocarcinoma patients with tumors expressing high or low levels of SIRT1 combined with high or low levels of ISG15 (*P* < 0.001). **i** A schematic model showing a novel molecular mechanism through which SIRT1 ISGylation controls tumor progression and sensitivity to DNA damage-based therapeutics in lung cancer. SIRT1 ISGylation impairs the association of SIRT1 with DBC1, thereby potentiating the enzymatic activity of SIRT1, which promotes tumorigenesis and limits the sensitivity of lung cancer to DNA damage-based therapeutics.

Prompted by our findings that SIRT1 ISGylation is important for tumor progression and the therapeutic response in lung cancer, we analyzed the protein expression levels of SIRT1 and ISG15 in eleven pairs of primary lung adenocarcinoma tissues and adjacent normal tissues. Higher expression of SIRT1 was found in seven of the lung cancer tissues (63.6%) than in the paired adjacent normal tissues (Fig. 7a). In line with our analyses of the CCLE and TCGA databases (Fig. 2a–c), the protein levels of ISG15 and ISGylation conjugates were high in eight of the lung cancer tissues (72.7%). Interestingly, the levels of not only SIRT1 but also ISG15 and protein ISGylation conjugates were elevated in six of the lung cancer tissues (54.5%), suggesting the involvement of ISG15 and SIRT1 in lung cancer development. The tumor microenvironment is the environment surrounding tumors, where cells continuously recognize danger and damage signals via extracellular and intracellular pattern recognition receptors (PRRs)^66^. One of the important events is type I IFN production and the subsequent upregulation of ISG15 and its related enzymes during cancer development, supporting our findings of elevated ISG15 expression and ISGylation in lung cancer tissues^2^ and suggesting that the upregulation of ISG15 expression and ISGylation is directly or indirectly linked to the pathogenesis of cancer.

To further extend our observations to a clinicopathologically relevant context, we analyzed the protein expression levels of SIRT1 and ISG15 in NSCLC patients-derived lung cancer tissues. After dual-immunohistochemical staining (IHC), SIRT1 was visible as brown staining and ISG15 as red staining (Fig. 7b). Based on the IHC staining scores (Supplementary Fig. 13), the lung cancer tissues were subdivided into high- and low-expression groups. According to these cutoff values, no clinicopathologic factor was significantly associated with the combined expression of SIRT1 and ISG15 (SIRT1/ISG15) or with the individual expression of SIRT1 or ISG15 (Supplementary Table 1). High SIRT1 expression (SIRT1^hi^ (log rank *P* = 0.028)) and high ISG15 expression (ISG15^hi^ (log rank *P* = 0.022)) were each significantly associated with a decrease in overall survival in NSCLC patients (Fig. 7c, d). Importantly, high combined expression of SIRT1 and ISG15 (SIRT1^hi^/ISG15^hi^ (log-rank *P* < 0.001)) was positively correlated with poor prognosis in NSCLC patients (Fig. 7e). Given that patients with lung adenocarcinoma had a 3.206-fold greater risk of death (95% CI: 1.293–7.947) than patients with squamous cell lung carcinoma, we performed an additional analysis to evaluate the prognostic significance of SIRT1, ISG15, and SIRT1/ISG15 expression in patients with squamous cell lung carcinoma and lung adenocarcinoma. SIRT1^hi^ (log rank *P* = 0.018) was associated with decreased overall survival in squamous cell lung carcinoma patients (Fig. 7f). Intriguingly, SIRT1^hi^/ISG15^hi^ was significantly correlated with poor prognosis in not only squamous cell lung carcinoma patients (log rank *P* = 0.012) (Fig. 7g) but also lung adenocarcinoma patients (log rank *P* < 0.001) (Fig. 7h). In univariate analysis, the expression status of SIRT1, ISG15, or SIRT1/ISG15 was identified as a factor strongly associated with overall survival (Supplementary Table 2). The SIRT1^hi^/ISG15^hi^ phenotype was associated with a 3.477-fold greater risk of death and poor survival (95% confidence interval (95% CI); 1.685–7.176, *P* < 0.001) in lung cancer patients compared with the SIRT1^lo^/ISG15^lo^ phenotype. According to our multivariate analysis, the SIRT1^hi^/ISG15^hi^ phenotype was an independent indicator of a shorter survival time in lung cancer patients (Supplementary Table 3). The SIRT1^hi^/ISG15^hi^ phenotype was associated with a 4.984-fold greater risk of death (95% CI: 2.175–11.423, *P* < 0.001) in patients. Taken together, our results revealed that elevated expression levels of SIRT1 and ISG15 are associated with the progressive phenotype of lung cancer and are positively correlated with poor prognosis in lung cancer patients, suggesting that ISG15 and SIRT1 are prognostic indicators in human lung cancer.

## Discussion

In response to both extracellular and intracellular perturbations, SIRT1 is tightly regulated by alterations in its expression, associations with other proteins, and posttranslational modifications (PTMs), which can ultimately govern physiological and pathophysiological processes and diseases. Although SIRT1 has been studied in cancer research in recent decades, the role of SIRT1 in cancer has remained controversial, likely due to the genetic background of cancer, type of tissue, stage of cancer, and distinct regulation of SIRT1, which might differentially affect substrates. Our current work reveals a novel molecular mechanism underlying SIRT1 ISGylation for the regulation of tumor progression and the response to DNA-damaging chemotherapeutics in lung cancer, as depicted in the summary schematic (Fig. 7i).

We showed that SIRT1 ISGylation induced by treatment with the DNA-damaging chemotherapeutic agent doxorubicin impairs the interaction of SIRT1 with DBC1, which unleashes SIRT1 from its inactive state and leads to an increase in its deacetylase activity. Although the structure for the complex between SIRT1 and DBC1 has not been determined, the ESA region located in the C-terminus of SIRT1 has been shown to compete with DBC1 to interact with the catalytic core domain^65^. Based on the ISGylation sites in SIRT1, we speculate that ISGylation at Lys444 might be involved in the dissociation of DBC1 to expose the substrate binding site. The functional consequence of ISGylation at Lys314 has not been determined. However, we are tempted to speculate that ISGylation at Lys314 induces a conformational change to a closed state, because the zinc-binding and helical modules in the catalytic domain move upon binding of the cosubstrate NAD^+^ and a substrate^67^. Additionally, it is possible that Lys519 may participate in strengthening the binding of the ESA to the catalytic core. However, the exact structural changes in SIRT1 caused by ISGylation need to be further explored via structural studies.

PTMs can occur very rapidly in cells and are highly dynamic to accommodate constantly changing signals in the cells. In addition to single regulatory PTMs, there are also PTMs that function in orchestrated manners. The combinatorial action of multiple PTMs on the same protein or different proteins is termed PTM crosstalk^68,69^. It is possible that ISGylation of SIRT1 might occur in concert or compete with multiple PTMs of SIRT1, thereby driving cumulative outputs to modulate the catalytic activity, binding affinity for substrate proteins, stability, and subcellular localization of SIRT1. The N- and C-terminal regulatory domains of SIRT1 are targets of numerous PTMs, including phosphorylation, SUMOylation, glycosylation, and S-glutathionylation^70–72^. SIRT1 is SUMOylated at a lysine residue in the C-terminus, preserving its deacetylase activity, which permits SIRT1 to inhibit the transcription of apoptosis-related genes^73^. PTMs in the catalytic core domain of SIRT1 have begun to be identified. Recently, phosphorylation, S-nitrosylation, and carbonylation have been demonstrated to regulate SIRT1^74–80^. SIRT1 was shown to be ubiquitinated in the catalytic core domain, which decreases its stability^81,82^. Therefore, future studies to explore how one dynamic PTM can affect another PTM in the same or a neighboring domain and how the mechanisms of crosstalk between PTMs can result in coordinated control of the activity of SIRT1 are likely worthwhile.

SIRT1 ISGylation promotes not only tumorigenesis but also chemoresistance in vivo and in vitro models of lung cancer. Lung cancer is the most fatal cancer worldwide, mainly because of the rapid emergence of intrinsic and acquired chemoresistance, adaptive oncogenic mutations, and poor prognosis^83^. Although several pathological parameters, including tumor-positive lymph node status and tumor size, have been suggested to have prognostic value in lung cancer patients, there are still limitations in predicting lung cancer progression, recurrence, and drug resistance. From the perspective of clinical relevance, we revealed that SIRT1 and ISG15 were upregulated in primary lung adenocarcinoma tissues from lung cancer patients compared to the corresponding adjacent normal tissues. Furthermore, elevated expression of SIRT1 and ISG15 in NSCLC tumors was strongly associated with poor prognosis, suggesting that SIRT1 ISGylation is of potential prognostic value in NSCLC patients. Elevated expression of SIRT1 in human NSCLCs is positively associated with advanced tumor stage, metastasis, and worse prognosis^84–86^. Importantly, SIRT1 upregulation is positively correlated with both a reduction in apoptosis and resistance to chemotherapeutic drugs, including doxorubicin, in various types of cancer and in cancer stem cells^87–91^. SIRT1 inhibition enhances the antitumor activity of MK-1775 in lung cancer^49^. Furthermore, SIRT1 is a potential predictor of poor prognosis in NSCLC patients treated with platinum-based chemotherapy, and SIRT1 downregulation greatly increases chemosensitivity to cisplatin, indicating the important role of SIRT1 in driving lung cancer development and chemoresistance. Therefore, our findings pave the way for understanding the regulatory mechanism of SIRT1 in cancer cell fate decisions. Moreover, our preliminary transcriptional profiles generated by RNA-Seq of cells derived from lung cancer with or without doxorubicin suggested that SIRT1 could be positively correlated with the expression of genes in key pathways associated with the propensity for cancer development and resistance to various therapies, including the mitogen-activated protein kinase (MAPK), phosphoinositide 3-kinase (PI3K)-protein kinase B (AKT), Ras, Janus kinase/signal transducer and activator of transcription (JAK-STAT), and nuclear factor kappa-light-chain-enhancer of activated B cells (NF-κB) signaling pathways (unpublished data). Further in-depth investigations of the transcriptomic features coordinated by SIRT1 and SIRT1 ISGylation might be geared toward not only understanding the contributions of SIRT1 and SIRT1 ISGylation to cancer cell proliferation and apoptosis but also identifying feasible biomarkers for therapeutic responsiveness.

Lysine acetylation is one of the most important PTMs and broadly regulates diverse sets of cellular functions. Therefore, understanding the dynamics of lysine acetylation is essential for deciphering its functions in the regulation of physiological processes and dysregulated or disease states. We are currently attempting to determine how changes in the lysine acetylome are regulated by SIRT1 ISGylation and to delineate the mechanism through which acetylome dynamics are implicated in tumorigenesis and the therapeutic response.

ISG15 has emerged as an important regulator of diverse cellular processes, including metabolic reprogramming, the immune response, the DNA damage response, autophagy, exosome secretion, cytoskeletal dynamics, and telomere shortening^2,31,32,53,92–96^. However, studies on the functions of ISG15 in cancer pathogenesis and therapeutic responsiveness have generated many conflicting results that indicate its context-dependent tumor-promoting or tumor-suppressive activity. Here, our current work highlights the importance of ISGylation in orchestrating lung cancer progression and limiting sensitivity to DNA damage-inducing therapy. This mechanism is highly consistent with the results in our in vivo model and clinical outcomes. Unlike the comparable protein levels of ISG15 and ISGylation conjugates between clonal cell lines (Fig. 4b), significant induction of protein ISGylation conjugates formation but only marginal induction of the expression of free ISG15 was observed in tumors in the presence of doxorubicin, potentially because of biological events occurring in vivo (Fig. 6c). Interestingly, the protein levels of ISG15 and/or ISGylation conjugates in tumors formed from SIRT1 WT-complemented cells were higher than those in tumors formed from SIRT1 KR-complemented cells in either the absence or presence of doxorubicin, which might explain why the tumors formed from SIRT1 WT-complemented cells in the absence of doxorubicin were larger than the tumors formed from SIRT1 KR-complemented cells, suggesting the necessity of further investigation of the relationship between SIRT1 ISGylation and the induction of ISG15 expression and protein ISGylation conjugates formation in vivo. Another question is why our in vivo findings did not precisely mirror our in vitro findings. ISG15 and protein ISGylation conjugates affect the physiological states of tumors in a manner dependent on internal and external stimuli and orchestrate tumor–tumor microenvironment communication during cancer development, resulting in tumor progression or tumor suppression^97^. These complex biological responses might result from the pleiotropic actions of ISG15 and ISGylation as well as various feedback control mechanisms, which appear to be dependent on the tumor context and tumor microenvironment. Therefore, we cannot rule out the possibility that the induction of ISG15 expression and protein ISGylation conjugates formation in lung tumors modulates the tumor environment, suggesting the necessity of further investigation of the role of ISG15 and ISGylation in the regulation of tumor–tumor microenvironment communication and the coordination of tumor responses to therapies.

Chemo- and radiotherapy are designed to eliminate cancer cells by inducing DNA damage exceeding the capacity of the DNA damage response^98^. However, cancer cells often exhibit abnormalities in the DNA damage response, rendering them resistant to DNA damage-based therapy^99^. In addition, even though cancer patients initially exhibit favorable responses to the widely used chemotherapeutic drug doxorubicin, the majority of cancer patients experience undesirable side effects and exhibit intrinsic or acquired resistance. *ISG15* is an IFN-related DNA damage resistance signature (IRDS) gene^56^. The IRDS is a signature indicating resistance to DNA-damaging therapies, suggesting that information about the IRDS status significantly improves outcome prediction when combined with standard markers, risk group stratification, or other genomic classifiers. Importantly, ISG15 has been demonstrated to promote resistance to DNA-damaging chemo- and radiotherapy in different cancer types and to be correlated with unfavorable prognosis^100^, suggesting the potential of targeting ISG15 for resensitization of tumor cells and improvement of the outcome of anticancer therapy. Overall, we envisage that targeting SIRT1 ISGylation could amplify the antitumor effects of DNA damage-based therapies in the treatment of lung cancer.

### Supplementary information

The supplementary information is provided in a PDF file. The supplementary information for this article is available at https://www.nature.com/emm and accompanies the manuscript on the Experimental & Molecular Medicine website (https://www.nature.com/emm).

### Supplementary information

Supplemental Information

## Data Availability

The data generated or analyzed during this study are included in this published article and its supplementary data files. All relevant data supporting the present study are available from the corresponding author upon reasonable request.
