# Supplementary material for: SIRT1 ISGylation accelerates tumor progression by unleashing SIRT1 from the inactive state to promote its deacetylase activity
Source: Exp Mol Med. 2024 Mar 5;56(3):656–73. doi: 10.1038/s12276-024-01194-2 (PMC10985095; doi:10.1038/s12276-024-01194-2)
Supplement: Supplementary file 1 — Supplemental Information [file 12276_2024_1194_MOESM1_ESM.pdf]

Supplementary Information

SIRT1 ISGylation accelerates tumor progression by unleashing SIRT1 from the inactive state to promote its deacetylase activity

Ji An Kang[1,2,3*], Yoon Jung Kim[1,2,3*], Kyu Yun Jang[4], Hye Won Moon[1,3], Haeseung Lee[5], Seonjeong Lee[6,7], Hyun Kyu Song[8], Sang Woo Cho[9], Yoon Sun Yoo[1,3], Hye Gyeong Han[1,3], Min-Ju Kim[5], Myoung Ja Chung[4], Cheol Yong Choi[9], Cheolju Lee[6,7], Chaeuk Chung[10], Gang Min Hur[11], You-Sun Kim[12], and Young Joo Jeon[1,3]

1. Supplementary Figures 1-13
2. Supplementary Tables 1-3

Proteins	Full name
AHCYL1	S-adenosylhomocysteine hydrolase-like 1
AIP	Aryl hydrocarbon receptor interacting protein
ALDOA	Aldolase A, fructose-bisphosphate
ANP32A|ANP32B|ANP32C|ANP32D	Acidic (leucine-rich) nuclear phosphoprotein 32 family, member; A or B or C or D
ANP32B	Acidic (leucine-rich) nuclear phosphoprotein 32 family, member B
ATP5A1	ATP synthase, H+ transporting, mitochondrial F1 complex, alpha
ATP5H	ATP synthase, H+ transporting, mitochondrial F0 complex, subunit d
CA1	Carbonic anhydrase I
CACYBP	Calcyclin binding protein
CSNK2B	Casein kinase 2, beta polypeptide
DDB1	Damage-specific DNA binding protein 1, 127kDa
DHX30	DEAH (Asp-Glu-Ala-His) box polypeptide 30
EWSR1	Ewing sarcoma breakpoint region 1; isoform unknown
FKBP5	FK506 binding protein 5
FUS	Fusion (involved in t(12;16) in malignant liposarcoma)
GAPDH	Glyceraldehyde-3-phosphate dehydrogenase
GNB2L1	Guanine nucleotide binding protein (G protein), beta polypeptide
GPRASP2	G protein-coupled receptor associated sorting protein 2
HSPE1	Heat shock 10kDa protein 1 (chaperonin 10)
IMP-1	Tumor suppressor candidate 4
IMPDH2	IMP (inosine monophosphate) dehydrogenase 2
KCTD17	Potassium channel tetramerisation domain containing 17
KCTD2	Potassium channel tetramerisation domain containing 2
KCTD5	Potassium channel tetramerisation domain containing 5
KIF21B	Kinesin family member 21B
KPNA1	Karyopherin alpha 1 (importin alpha 5)
LDHA	Lactate dehydrogenase A
LDHB	Lactate dehydrogenase B
NDUFA2	NADH dehydrogenase (ubiquinone) 1 alpha subcomplex, 2, 8kDa
PABPC1	Poly(A) binding protein, cytoplasmic 1
PABPC3|PABPC1	Poly(A) binding protein, cytoplasmic; 3 or 1
PAICS	Phosphoribosylaminoimidazole carboxylase, phosphoribosylaminoimidazole succinoca rboxamide synthetase
PCDHGC3|PCDHGC4	Protocadherin gamma subfamily C; 3 or 4 (isoform unknown)
PCNA	Proliferating cell nuclear antigen
PHB2	Prohibitin 2
PHGDH	Phosphoglycerate dehydrogenase
PKM2	Pyruvate kinase, muscle; isoform unknown
PPM1G	Protein phosphatase 1G (formerly 2C), magnesium-dependent, gamma
PRDX2	Peroxiredoxin 2; isoform unknown
PRDX4	Peroxiredoxin 4
PRKD2|PRKD1|PRKD3	Protein kinase; D2 or D1 or D3
RCN2	Reticulocalbin 2, EF-hand calcium binding domain
RUVBL2	RuvB-like 2 (E. coli)
SART3	Squamous cell carcinoma antigen recognised by T cells 3
SDCCAG3	Serologically defined colon cancer antigen 3
SERBP1	SERPINE1 mRNA binding protein 1
**SIRT1**	**Sirtuin (silent mating type information regulation 2 homolog) 1 (S. cerevisiae)**
SLC39A7	Solute carrier family 39 (zinc transporter), member 7
STAT1	Signal transducer and activator of transcription 1, 91kDa; isoform unknown
STAT2	Signal transducer and activator of transcription 2, 113kDa
TJP2	Tight junction protein 2 (zona occludens 2); isoform unknown
TRA1	Tumor rejection antigen (gp96) 1
UBC|RPS27A|UBA52|UBB	Ubiquitin family
UBE1	Ubiquitin-activating enzyme E1
UBE2C	Ubiquitin-conjugating enzyme E2C; isoform unknown
UBE2NL|UBE2N	Ubiquitin-conjugating enzyme; E2N-like or E2N (UBC13 homolog, yeast)
VDAC1	Voltage-dependent anion channel 1
VDAC2	Voltage-dependent anion channel 2
VDAC4|VDAC1|VDAC3	Voltage-dependent anion channel; 4 or 1 or 3
XRCC6	X-ray repair complementing defective repair in Chinese hamster cells

Supplementary Fig. 1. Related to Fig. 1. ISG15-conjugating system was expressed in HEK293T cells and their lysates were prepared and incubated with anti-Flag M2 affinity gel in which anti-Flag antibody is covalently attached to agarose. Bound proteins were eluted with Flag peptide and subjected to LC-MS/MS analysis.

>sp|Q96EB6|SIR1_HUMAN NAD-dependent protein deacetylase sirtuin-1 GN=SIRT1

MADEAALALQPGGSPSAAGADREAASSPAGEPLRKRPRRDGPGLER**SPGEPGGAAPER**EV

PAAAR**GCPGAAAAALWREAEAEAAAAGGEQEAQATAAAGEGDNGPGLQGPSREPPLADNL**

YDEDDDDEGEEEEEAAAAAIGYRDNLLFGDEIITNGFHSCESDEEDR**ASHASSSDWTPRP**

R**IGPYTFVQQHLMIGTDPR**TILK**DLLPETIPPPELDDMTLWQIVINILSEPPKR**KK**RKDI**

NTIEDAVKLLQECK**KIIVLTGAGVSVSCGIPDFR**SRDGIYAR**LAVDFPDLPDPQAMFDIE**

YFRKDPRPFFK**FAKEIYPGQFQPSLCHK**FIALSDKEGKLLR**NYTQNIDTLEQVAGIQRII**

QCHGSFATASCLICKYKVDCEAVR**GDIFNQVVPR**CPR**CPADEPLAIMKPEIVFFGENLPE**

QFHRAMKYDKDEVDLLIVIGSSLKVRPVALIPSSIPHEVPQILINREPLPHLHFDVELLG

DCDVIINELCHRLGGEYAKLCCNPVK**LSEITEKPPR**TQK**ELAYLSELPPTPLHVSEDSSS**

PERTSPPDSSVIVTLLDQAAKSNDDLDVSESKGCMEEKPQEVQTSRNVESIAEQMENPDL

KNVGSSTGEKNERTSVAGTVRKCWPNRVAKEQISR**RLDGNQYLFLPPNR**YIFHGAEVYSD

SEDDVLSSSSCGSNSDSGTCQSPSLEEPMEDESEIEEFYNGLEDEPDVPER**AGGAGFGTD**

GDDQEAINEAISVKQEVTDMNYPSNKS

Supplementary Fig. 2. Related to Fig. 1. The sequence coverage of SIRT1 by mass spectrometry and GlyGly-modified lysine sites. The sequences in bold and underlined correspond to the peptides identified by mass spectrometry. The lysine residues identified as being GlyGly-modified by mass spectrometry are highlighted in red.

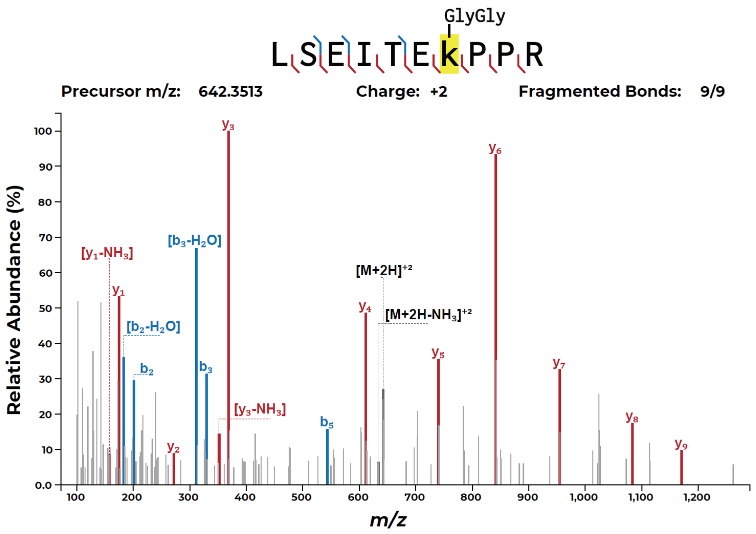

Supplementary Fig. 3. Related to Fig. 1. The annotated tandem mass (MS/MS) spectra. Annotated MS/MS spectrum of the peptide, LSEITEKPPR from SIRT1. The Lys residue highlighted with yellow color is at position 513 in SIRT1 and is GlyGly-modified.

>sp|P05161|ISG15_HUMAN Ubiquitin-like protein ISG15 GN=ISG15

MGWDLTVKMLAGNEFQVSLSSSMSVSELKAQITQKIGVHAFQQR**LAVHPSGVALQDRVPL**

ASQGLGPGSTVLLVVDKCDEPLSILVRNNKGRSSTYEVRLTQTVAHLKQQVSGLEGVQDD

LFWLTFEGKPLEDQLPLGEYGLKPLSTVFMNLRLRGGGTEPGGRS

Supplementary Fig. 4. Related to Fig. 1. The sequence coverage of ISG15 by mass spectrometry. The sequences in bold and underlined correspond to the peptides identified by mass spectrometry.

Lys position	Peptide sequence	Abundance based on LFQ
K314	FAkEIYPGQFQPSLcHK	1.10E+08
	EIYPGQFQPSLcHK	4.00E+09
K513	LSEITEkPPR	2.04E+07
	LSEITEKPPR	4.33E+09

Supplementary Fig. 5. Related to Fig. 1. Abundances of the peptides based on label-free quantification (LFQ) approach. The lysine in red and lowercase is the site found to be GlyGly-modified. The abundance value is calculated based on the precursor peak area.

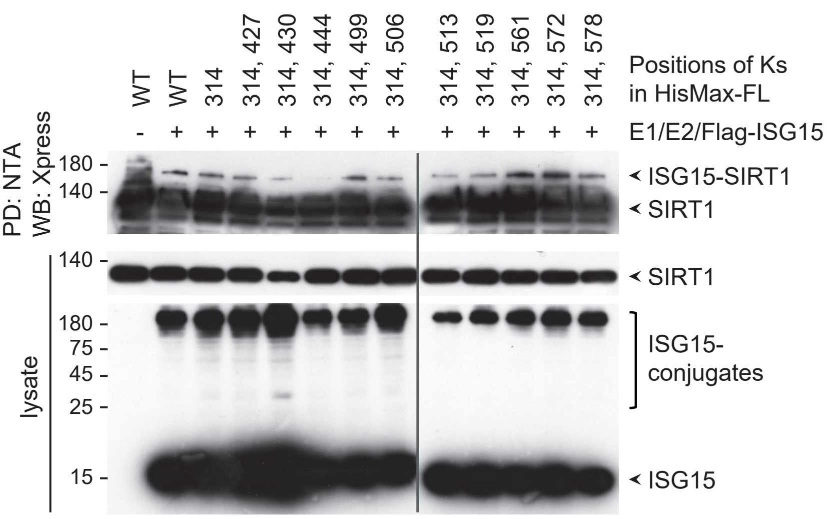

Supplementary Fig. 6. Related to Fig. 1. Full-length SIRT1 (HisMax-FL) and K-to-R mutants were expressed in HEK293T cells with ISG15-conjugating system. Cell lysates were subjected to PD with NTA resins followed by WB with anti-Xpress antibody. The lysates were also directly probed with indicated antibodies.

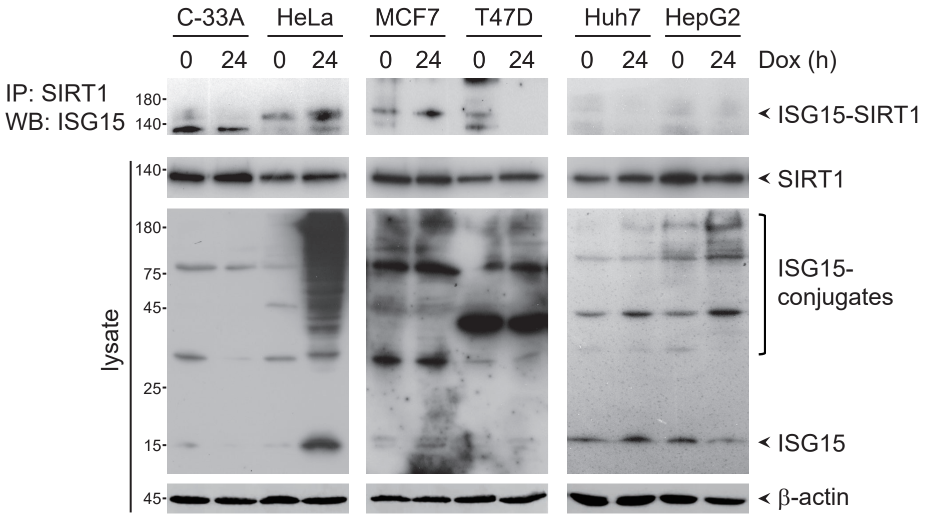

Supplementary Fig. 7. Related to Fig. 3. C-33A, HeLa, MCF7, T47D, Huh7, and HepG2 cells incubated with doxorubicin were subjected to IP with anti-SIRT1 antibody followed by WB with anti-ISG15 antibody.

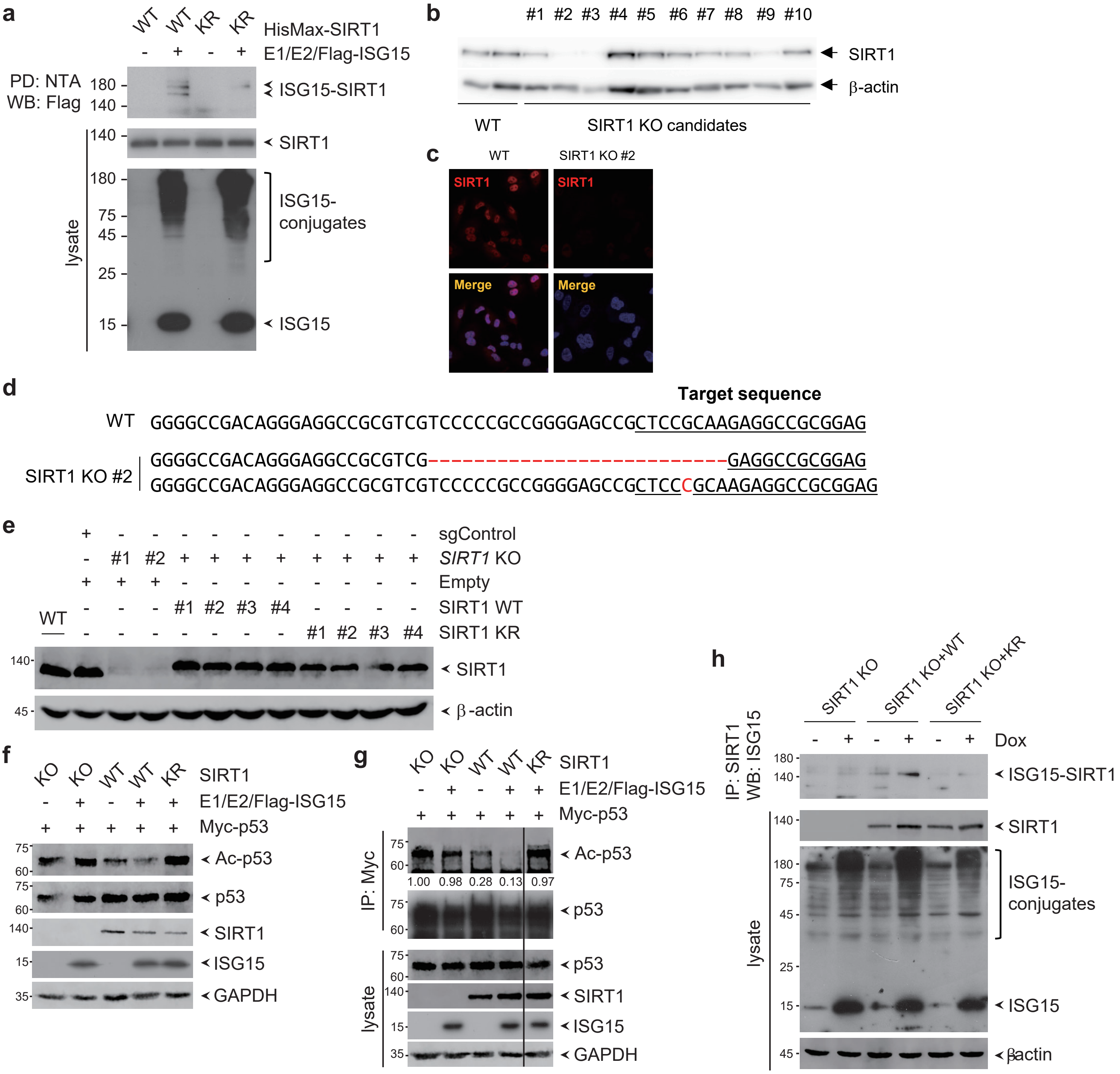

Supplementary Fig. 8. Related to Fig. 4. a Related to Fig. 4a, lysates from the cells transfected as in Fig. 4a were subjected to PD with NTA resins followed by WB with anti-Flag antibody. **b** Cell lysates from SIRT1 knockout clonal A549 cells (SIRT1 KO) after CRISPR-based gene editing with SIRT1 sgRNA or parental A549 cells (WT) were subjected to WB with anti-SIRT1 antibody. **c, d** Immunocytochemistry (c) and genomic DNA sequencing (d) analyses for SIRT1 in WT and clone #2 SIRT1 KO cells. 26-nt deletion and 1-nt insertion are able to shift the reading frame of SIRT1 and inactivate SIRT1. **e** sgControl-expressing A549 cells or SIRT1 KO cells were stably complemented with SIRT1 WT, SIRT1 KR or lentiviral control vector. Clonal cell lines were selected. Cell lysates were subjected to WB with indicated antibodies. **f, g** Myc-tagged p53 was transiently expressed with or without ISG15-conjugating system in SIRT1 KO cells, SIRT1 WT- or SIRT1 KR-complemented cells. Cell lysates were subjected to WB with indicated antibodies (f) or subjected to IP with anti-Myc antibody followed by WB with anti-Acetyl-p53 antibody (g). The band intensities were determined by using a densitometer and normalized by those of immunoprecipitated p53. The normalized densities for Ac-p53 seen with SIRT1 KO cells in the absence of ISG15-conjugating system were expressed as 1.00 and the others were as its relative values. **h** Related to Fig. 4g, lysates from the cells treated as in Fig. 4g were subjected to IP with anti-SIRT1 antibody followed by WB with anti-ISG15 antibody.

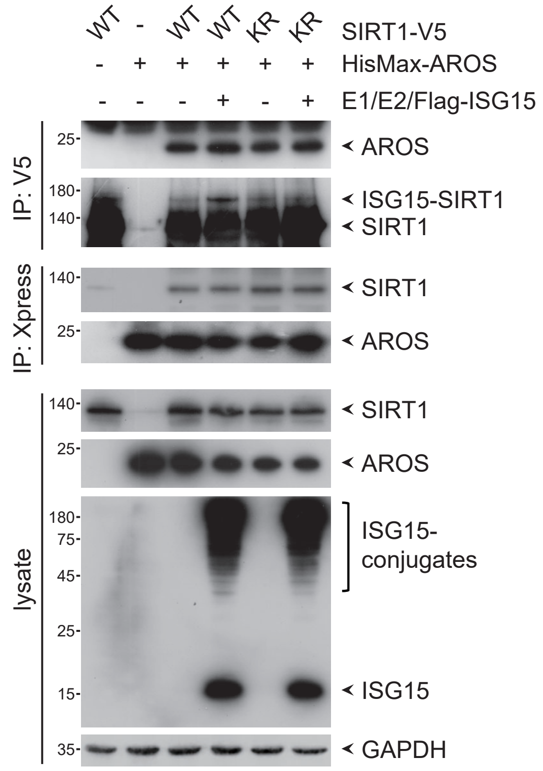

Supplementary Fig. 9. Related to Fig. 4. V5-tagged SIRT1 WT or SIRT1 KR was transiently co-expressed with HisMax-tagged AROS with or without ISG15-conjugating system. Cell lysates were subjected to IP with anti-V5 or anti-Xpress antibody followed by WB with anti-Xpress or anti-V5 antibody.

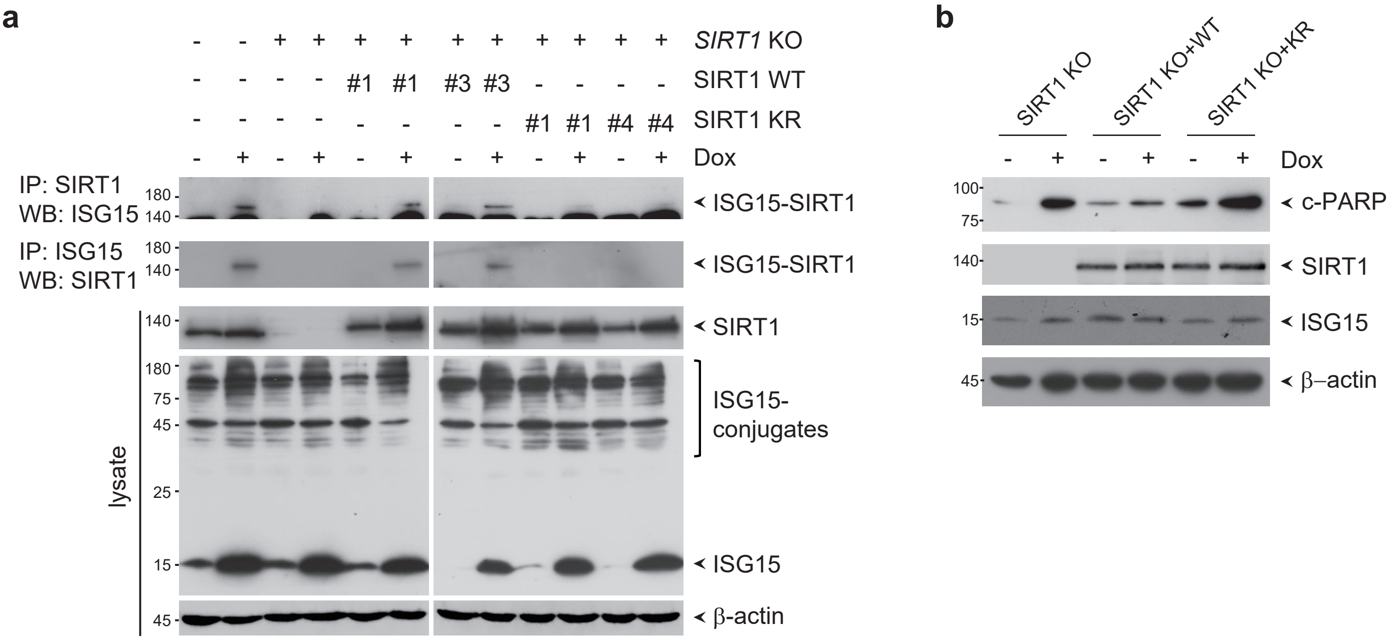

Supplementary Fig. 10. Related to Fig. 5. a Cell lysates used in cell counting (Fig. 5a) were subjected to IP with anti-SIRT1 or anti-ISG15 antibody followed by WB with anti-ISG15 or anti-SIRT1 antibody. **b** SIRT1 KO cells or SIRT1 KO cells complemented with SIRT1 WT or SIRT1 KR were incubated 0.5 μM doxorubicin for 36 hours. Cell lysates were subjected to WB with indicated antibodies.

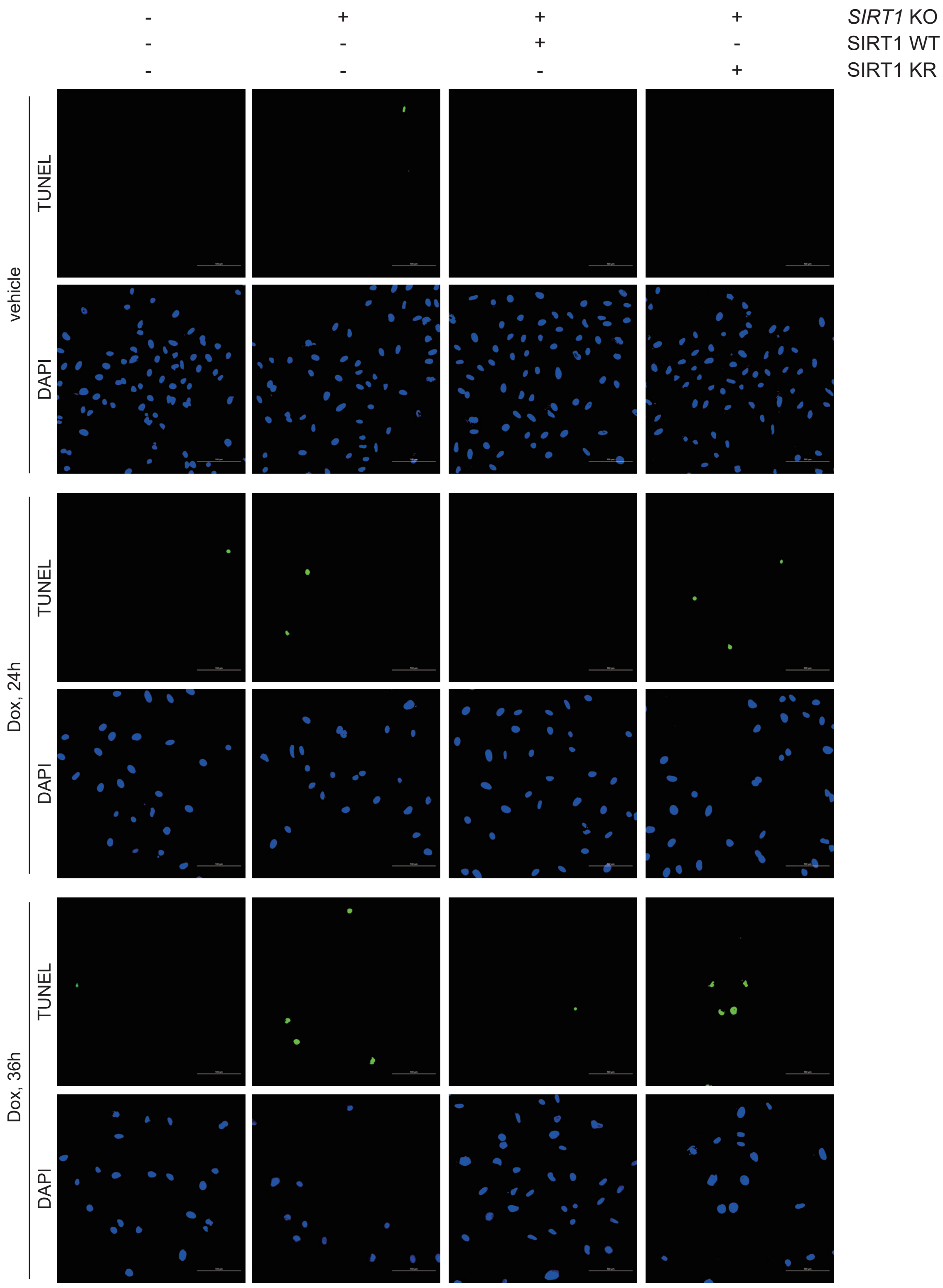

Supplementary Fig. 11. Related to Fig. 5. SIRT1 KO, SIRT1 KO cells complemented with SIRT1 WT or SIRT1 KR, and Control cells were incubated with 1 μM doxorubicin for indicated times. They were then subjected to TUNEL assay. Scale bars, 100 μm.

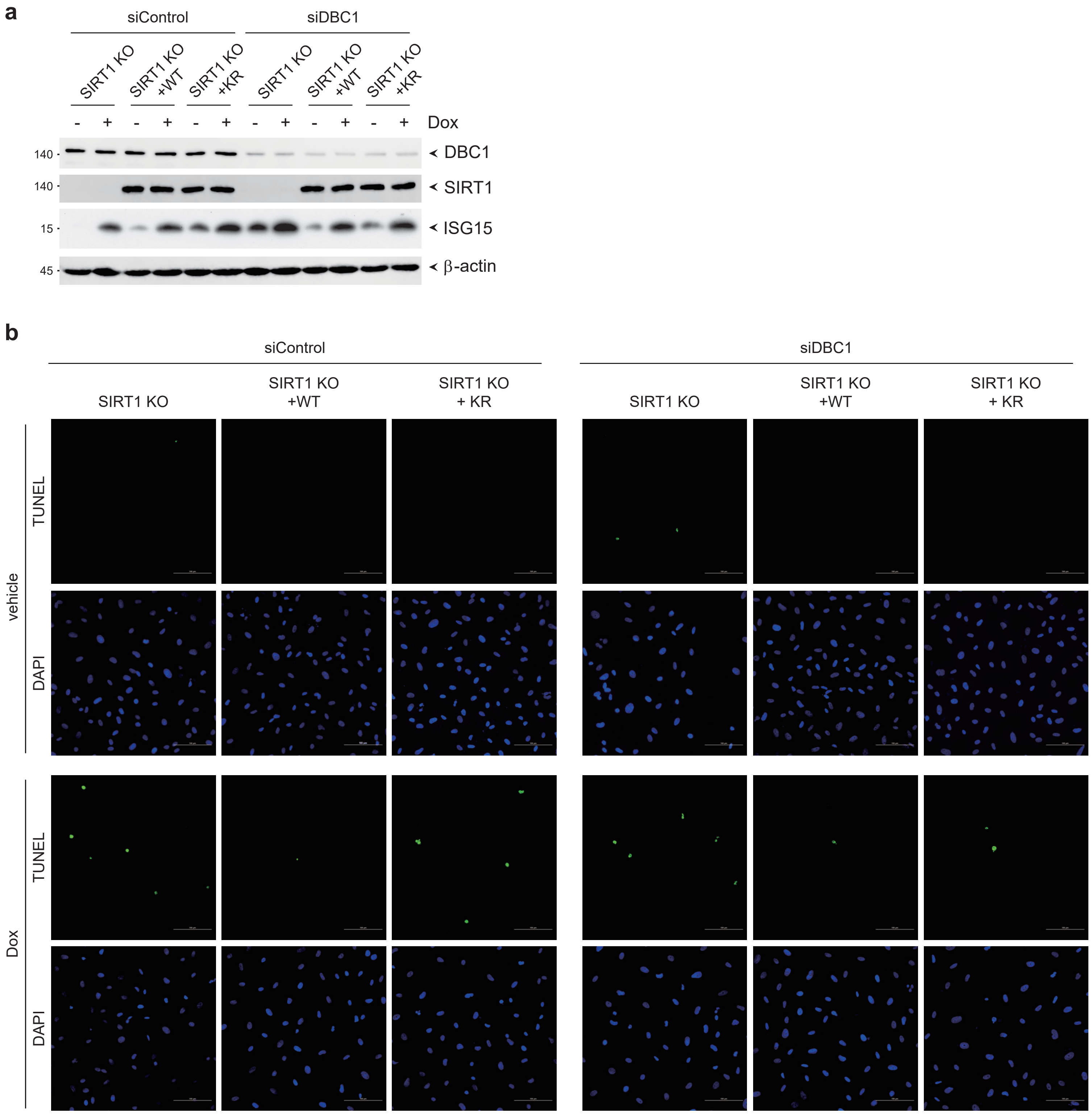

Supplementary Fig. 12. Related to Fig. 5. a, b SIRT1 KO or SIRT1 KO cells complemented with SIRT1 WT or SIRT1 KR were transfected with siControl or siDBC1 and incubated with 1 μM doxorubicin for 24 hours. They were then subjected to WB (a) or TUNEL assay (b). Scale bars, 100 μm.

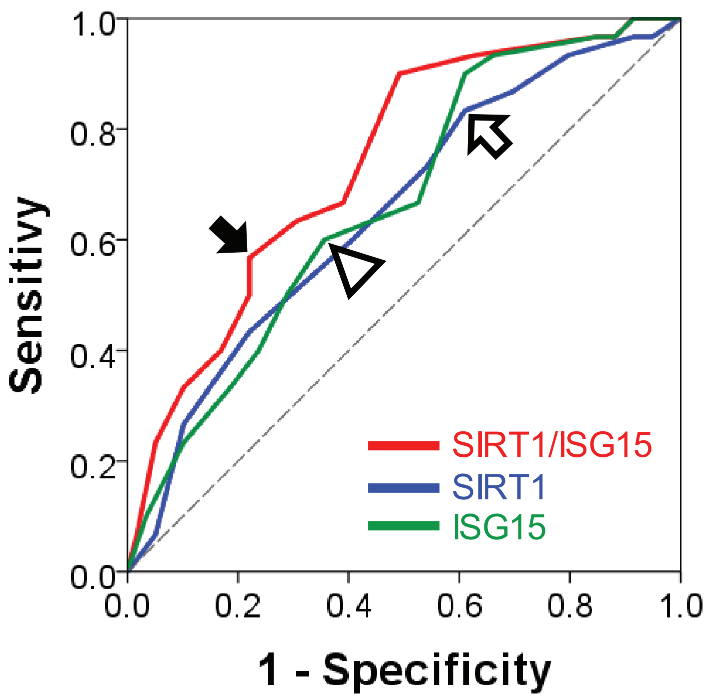

	Cut-off	AUC	*P*
SIRT1/ISG15	All	0.738	< 0.001
	≥ 11	0.673	0.008
SIRT1	All	0.651	0.021
	≥ 11	0.612	0.087
ISG15	All	0.664	0.012
	≥ 11	0.622	0.061

Supplementary Fig. 13. Related to Fig. 7. Receiver operating characteristic curve analysis to determine cut-off points for the immunohistochemical staining scores for co-expression of SIRT1 and ISG15 (SIRT1/ISG15, arrow), SIRT1 (empty arrow), or ISG15 expression (empty arrowhead).

Supplementary Table 1. Clinicopathologic variables, individuals, and expression pattern of SIRT1 and ISG15 in lung cancer patients

Table 1. Clinicopathologic variables, individual and expression pattern of SIRT1 and ISG15 in lung cancer patients

Characteristics		No.	SIRT1/ISG15		SIRT1		ISG15	
			Positive	*P*	Positive	*P*	Positive	*P*
Sex	Male	60	19 (32%)	0.558	40 (67%)	0.584	27 (45%)	0.747
	Female	29	11 (38%)		21 (72%)		12 (41%)	
Age, yrs	≤ 65	45	19 (42%)	0.086	34 (76%)	0.149	24 (53%)	0.067
	> 65	44	11 (25%)		27 (61%)		15 (34%)	
Smoking	Never smoker	51	15 (29%)	0.321	34 (67%)	0.659	19 (37%)	0.148
	Smoker	38	15 (39%)		27 (71%)		20 (53%)	
TNM stage	I & II	70	22 (31%)	0.383	48 (69%)	0.99	29 (41%)	0.383
	III & IV	19	8 (42%)		13 (68%)		10 (53%)	
T category	1 & 2	69	23 (33%)	0.89	46 (67%)	0.48	30 (43%)	0.904
	3 & 4	20	7 (35%)		15 (75%)		9 (45%)	
N category	0	60	18 (30%)	0.287	40 (67%)	0.584	24 (40%)	0.296
	1 & 2	29	12 (41%)		21 (72%)		15 (52%)	
Histologic type	SqCC	56	20 (36%)	0.114	40 (71%)	0.185	24 (43%)	0.434
	Adenocarcinoma	29	7 (24%)		17 (59%)		12 (41%)	
	Other	4	3 (75%)		4 (100%)		3 (75%)	
Histologic grade	WD & MD	74	24 (32%)	0.572	51 (69%)	0.864	33 (45%)	0.744
	PD	15	6 (40%)		10 (67%)		6 (40%)	
ISG15	Low	50	0 (0%)	< 0.001	31 (62%)	0.133		
	High	39	30 (77%)		30 (77%)			
SIRT1	Low	28	0 (0%)	< 0.001				
	High	61	30 (49%)					

Abbreviations: SIRT1/ISG15, co-expression of SIRT1 and ISG15; SqCC, squamous cell carcinoma; WD, well differentiated; MD, moderately differentiated; PD, poorly differentiated;

Supplementary Table 2. Univariate Cox proportional harzards regression analysis for overall survival in lung cancer patients

Table 2. Univariate Cox proportional hazards regression analysis for overall survival in lung cancer patients

Characteristics	No.	Hazard ratio	95% confidence interval	*P*
Sex, male (*vs.* female)	60/89	2.878	1.101-7.523	0.031
Age, yrs, > 65 (*vs.* ≤ 65)	44/89	1.421	0.690-2.925	0.340
Smoking, smoker *vs.* never smoker)	38/89	6.187	2.643-14.481	< 0.001
TNM stage, II & IV (*vs.* I & II)	19/89	5.032	2.427-10.434	< 0.001
T category, 3 & 4 (*vs.* 1 & 2)	20/89	2.491	1.183-5.245	0.016
N category, 1& 2 (*vs.* 0)	29/89	3.105	1.510-6.383	0.002
Histologic type, SqCC	56/89	1		0.006
Adenocarcinoma	29/89	3.321	1.568-7.033	0.002
Other	4/89	3.304	0.738-14.803	0.118
Histologic grade, PD (*vs.* WD& MD)	15/89	1.805	0.774-4.212	0.172
SIRT1/ISG15, High (*vs.* low)	30/89	3.477	1.685-7.176	< 0.001
SIRT1, High (*vs.* low)	39/89	2.785	1.065-7.282	0.037
ISG15, High (*vs.* low)	61/89	2.282	1.098-4.739	0.027

Abbreviations: SqCC, squamous cell carcinoma; WD, well differentiated; MD, moderately differentiated; PD, poorly differentiated; SIRT1/ISG15, co-expression of SIRT1 and ISG15

Supplementary Table 3. Multivariate Cox proportional harzards regression analysis for overall survival in lung cancer patients

Table 3. Multivariate Cox proportional hazards regression analysis for overall survival in lung cancer patients

Characteristics	Hazard ratio	95% confidence interval	*P*
Model 1*			
Smoking, smoker (*vs.* never smoker)	2.860	1.099-7.441	0.031
TNM stage, II & IV (*vs.* I & II)	3.269	1.471-7.266	0.004
Histologic type, SqCC	1		0.013
Adenocarcinoma	3.698	1.542-8.870	0.003
Other	1.611	0.340-7.623	0.548
SIRT1/ISG15, High (*vs.* low)	4.984	2.175-11.423	< 0.001
Model 2**			
Smoking, smoker (*vs.* never smoker)	4.826	2.003-11.627	< 0.001
TNM stage, II & IV (*vs.* I & II)	3.854	1.775-8.368	< 0.001
SIRT1, High (*vs.* low)	3.286	1.240-8.705	0.017

Abbreviations: SqCC, squamous cell carcinoma; WD, well differentiated; MD, moderately differentiated; PD, poorly differentiated; SIRT1/ISG15, co-expression of SIRT1 and ISG15. * Variables considered in multivariate analysis Model 1 were sex, smoking history, TNM stage, T category of tumor stage, N category of tumor stage, histologic type of cancers, co-expression pattern of SIRT1 and ISG15 (SIRT1/ISG15), and individual expressions of SIRT1 and ISG15. ** Variables considered in multivariate analysis Model 2 were sex, smoking history, TNM stage, T category of tumor stage, N category of tumor stage, histologic type of cancers, co-expression pattern of SIRT1 and ISG15 (SIRT1/ISG15), and individual expressions of SIRT1 and ISG15.
